# *In Vivo* Bioconcentration of 10 Anionic Surfactants in Rainbow Trout Explained by *In Vitro* Data on Partitioning and S9 Clearance

**DOI:** 10.1021/acs.est.1c05543

**Published:** 2022-04-25

**Authors:** Anton Ribbenstedt, James M. Armitage, Felix Günther, Jon A. Arnot, Steven T. J. Droge, Michael S. McLachlan

**Affiliations:** † Department of Environmental Science, Stockholm University, 106 91 Stockholm, Sweden; ‡ AES Armitage Environmental Sciences, Inc., Ottawa, Ontario K1L 8C3, Canada; § Department of Mathematics, Stockholm University, 106 91 Stockholm, Sweden; ∥ ARC Arnot Research and Consulting Inc., Toronto, Ontario M4M 1W4, Canada; ⊥ Department of Physical and Environmental Sciences, University of Toronto Scarborough, Toronto, Ontario M1C 1A4, Canada; # Institute for Biodiversity and Ecosystem Dynamics (IBED), University of Amsterdam, 1090 GE Amsterdam, The Netherlands

**Keywords:** BCF, kinetics, biotransformation, membrane lipid, IVIVE

## Abstract

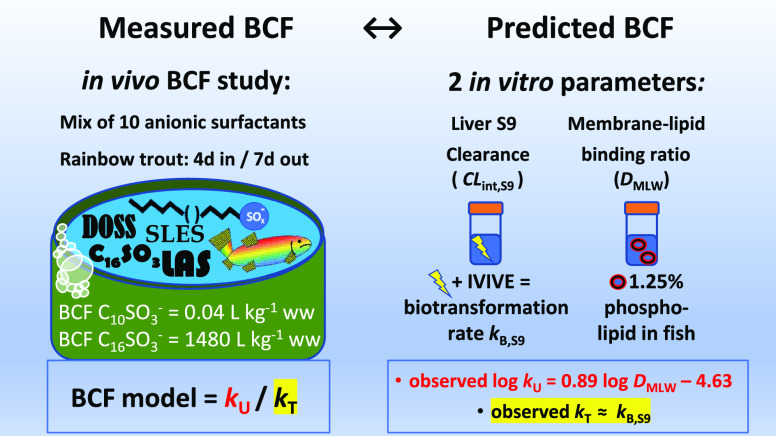

Bioconcentration factors (BCFs) in rainbow trout were measured for 10 anionic surfactants with a range of alkyl chain lengths and different polar head groups. The BCFs ranged from 0.04 L kg^–1^ ww (for C_10_SO_3_) to 1370 L kg^–1^ ww (C_16_SO_3_). There was a strong correlation between the log BCF and log membrane lipid–water distribution ratio (*D*_MLW_, *r*^2^ = 0.96), and biotransformation was identified as the dominant elimination mechanism. The strong positive influence of *D*_MLW_ on BCF was attributed to two phenomena: (i) increased partitioning from water into the epithelial membrane of the gill, leading to more rapid diffusion across this barrier and more rapid uptake, and (ii) increased sequestration of the surfactant body burden into membranes and other body tissues, resulting in lower freely dissolved concentrations available for biotransformation. Estimated whole-body *in vivo* biotransformation rate constants *k*_B-BCF_ are within a factor three of rate constants estimated from S9 *in vitro* assays for six of the eight test chemicals for which *k*_B-BCF_ could be determined. A model-based assessment indicated that the hepatic clearance rate of freely dissolved chemicals was similar for the studied surfactants. The dataset will be useful for evaluation of *in silico* and *in vitro* methods to assess bioaccumulation.

## Abstract 2

Membrane lipid−water partitioning governs both gill uptake and biotransformation of anionic surfactants in fish.

## Introduction

Anionic surfactants are widely used in products such as cleaning agents, detergents, and personal care products. Usage is high, with widespread professional and consumer use in Europe amounting to 97 889 tonnes year^–1^ in 2016 for alkyl sulfate surfactants alone.^1^ Anionic surfactants consist of a negatively charged functional group, frequently a sulfate or sulfonate, coupled with one or several linear or branched alkyl chains, which can also contain other groups. The range of structures is large; 45 anionic surfactants with production volumes >100 tonnes year^–1^ (of which 7 substances with 10 000–100 000 tonnes year^–1^) have been registered under the REACH legislation in the European Union (Table S1, Supporting Information). Important groups of anionic surfactants include alkyl sulfates (primarily used in detergents, production in North America 56 000 tonnes in 2006), alkylethoxysulfates (cleaning products, production in NA 229 000 tonnes in 2008), and linear alkyl benzene sulfonates (LASs) (detergents, production in NA 269 000 tonnes in 2008).^2^ Due to their extensive usage in down-the-drain products, emissions to surface water can be significant. Hence, careful evaluation of the potential environmental impact of anionic surfactants is required.

One important component of the regulatory assessment of the environmental safety of chemicals is bioaccumulation. Bioaccumulation is commonly evaluated using the bioconcentration factor [BCF, L kg^–1^ wet weight (ww)] in fish, which is derived from an experiment in which fish are exposed to the chemical via water, and defined as the quotient of the chemical’s concentration in fish and in water at steady state.^3^ Despite their low p*K*_a_ (typically < 0), anionic surfactants are taken up systemically by fish to a much greater extent than permanently charged cationic surfactants.^4,5^ BCFs were measured for a range of surfactants in the 1970s and 1980s. However, a review of the available data in 1994 concluded “Most of these data are inappropriate to quantitatively describe the bioconcentration of surfactants because the most frequently used analytical method, LSC without prior chromatographic separation of radiolabeled compounds, does not allow to distinguish between the parent compound and metabolites”.^6^ In a second review from 2007, 38 measured BCF values for anionic surfactants representing 10 C_10_–C_13_ LASs and 7 perfluoroalkyl acids were judged to be quantitatively reliable.^7^ There have since been several other BCF values published for LASs.^8−10^ Furthermore, BCF values from several unpublished industry studies have been reported, whereby few details are available on the methodology employed.^11^ Among the 45 anionic surfactants with a production volume >100 tonnes year^–1^ registered under REACH, only seven report *in vivo* BCF data (Table S1). In summary, the available quantitative BCF data for anionic surfactants are largely restricted to LAS; there is no consistent dataset for a range of anionic surfactant structures.

BCF tests require many animals.^3^ To reduce the usage of experimental animals, there are ongoing efforts to develop methods that can reliably predict BCF from *in vitro* tests and *in silico* models.^12−14^*In vitro* tests are used to measure biotransformation rates in cells or cell extracts, *in vitro in vivo* extrapolation (IVIVE) with *in silico* tools convert the *in vitro* data to hepatic and whole-body biotransformation clearance rates, and *in silico* bioconcentration models employ this information combined with the chemical’s partitioning properties to estimate the BCF.^15−17^ Anionic surfactants may be particularly amenable to this alternative approach because there are good methods for precise determination of the membrane lipid–water distribution ratio (*D*_MLW_) as the most relevant partitioning property,^18,19^ and there is evidence that their elimination is dominated by biotransformation,^8,10,20^ which would mean that only this elimination pathway would have to be considered. However, before an alternative approach for determining BCF can be implemented, it must be tested against *in vivo* BCF data.

The goal of this study was to determine *in vivo* BCFs of anionic surfactants that could be used to evaluate and further develop alternative approaches to estimate this metric for this class of chemicals. Considering this goal, particular attention was directed to selecting test chemicals that could provide insight into how molecular structure influences BCFs and to optimizing the experimental protocol to allow the maximum number of chemicals to be studied while ensuring that the BCF data were of sufficient quality for the stated purpose. For this, we drew on experience from earlier experiments on the tissue distribution and bioconcentration of cationic surfactants in rainbow trout.^5,21^ Furthermore, to supplement the interpretation of the *in vivo* data, a rainbow trout liver S9 substrate depletion assay (RT-S9) was used to measure *in vitro* intrinsic clearance rates of the same set of anionic surfactants.

## Methods

### Overview

The bioconcentration of a mixture of 10 anionic surfactants was measured in rainbow trout in water at pH 7.8. The S9 assays were conducted using both individual compounds and a mixture comparable to that used in the BCF experiment to explore the influence of co-solutes on biotransformation.

### Test Chemicals and Reagents

The test chemicals for the *in vivo* experiment were selected with a view to evaluating the influence of the alkyl chain length and the polar head group on the anionic surfactant BCF. Widespread use in household cleaning products and detergents was an exclusion criterion to minimize the risk of background contamination. The chemicals selected consisted of five linear alkyl sulfonates with chain lengths ranging from 10 to 16, four other linear alkyl surfactants with different anionic head groups [two sulfates, one LAS, one tetraethoxy sulfate (SLES)], and two more complex structures with two branched alkyl chains [bis(2-ethylhexyl)phosphate (BEHP) and bis(2-ethylhexyl)sulfosuccinate (DOSS)]. BEHP could not be reliably quantified in the BCF experiment, leaving 10 test chemicals in the study. The names and abbreviations of the test chemicals are provided in Table 1, while further information including CAS# and supplier are found in Table S2. Analytical standards were prepared in methanol and stored in glass. Polypropylene vials were employed for storing all extracts and solutions. The solvents employed are detailed in Table S3.

**Table 1:** Test Chemical Concentrations in Water (μg L^–1^) during the Exposure Phase: Nominal, Mean Observed, and Ratio Observed/Nominal^a^

abbreviation	name	nominal concn (μg L^–1^)	observed concn (μg L^–1^)	observed/nominal (%)	RSD (%)
C_10_SO_3_	decylsulfonate	52	63	120	8
C_11_SO_3_	undecylsulfonate	38	43	112	7
C_13_SO_3_	tridecylsulfonate	5.9	6.6	113	10
C_14_SO_3_	tetradecylsulfonate	5.6	7.6	136	12
C_16_SO_3_	hexadecylsulfonate	9.9	5.8	59	18
C_11_SO_4_	undecylsulfate	22	24	109	10
C_13_SO_4_	tridecylsulfate	7.1	11.9	166	15
C_10_-1-LAS	1-*n*-(*p*-sulfophenyl)decane	6.9	8.1	117	11
C_12_-EO_4_-SO_4_	dodecyltetraethoxysulfate	25	44	176	17
DOSS	bis(2-ethylhexyl)sulfosuccinate	26	54	204	22

a Mean and relative standard deviation (RSD).

### Fish Exposure and Sampling

The experiment was conducted in flow-through aquaria with juvenile rainbow trout at 10 °C. Ethical approval for the experiments was obtained from Stockholms djurförsöksetiska nämnd (decision 9967-2017). The fish, fish housing, fish handling, and procedure for introducing the mixture of 11 chemicals were very similar to our previous study with cationic surfactants;^5^ details are provided in Text S1. The experiment consisted of a 4-day exposure phase and a 7-day depuration phase. The nominal concentrations of the chemicals in water were chosen to minimize exposure while ensuring quantifiable concentrations in the fish based on the results of a pre-experiment and ranged from 5.6 to 52 μg L^–1^ (Table 1). Water samples were collected and fish were sacrificed according to the schedule in Table S4.

The water samples were collected just prior to the daily removal of feces from the aquarium. Triplicate samples were taken at each time point. An autopipette with a polypropylene tip was pumped 7 times with aquarium water, and then 400 μL of aquarium water was sampled and transferred to a 1.5 mL polypropylene vial containing 600 μL of methanol and isotope-labeled standards of BEHP and sodium dodecylsulfate (C_12_SO_4_). The water/methanol mixture was analyzed using liquid chromatography–mass spectrometry (LC–MS)/MS as described below for fish.

At each fish sampling, five fish were sacrificed. Following stunning and severance of the spinal cord, the whole fish were placed in a polyethylene bag, weighed, and immediately frozen at −20 °C. The weight of the individual fish at sacrifice was 24.0 ± 5.6 g.

### Sample Analysis

Three of the five fish from each time point were prioritized for analysis based on proximity to the median fish weight. Each fish was semithawed and homogenized with an Ultra-turrax dispersing device. For extraction, 0.15 mL of internal standard solution [isotope-labeled standards of BEHP (D_34_) and C_12_SO_4_ (D_25_)], 3 mL of methanol, and 3 steel balls (3.2 mm diameter) were added to 220 mg of the fish homogenate. This mixture was homogenized in a bullet blender for 5 min and then placed in an ultrasound bath at 45 °C for 15 min. Following centrifugation for 15 min@3200 RCF, the supernatant was decanted and the extraction was repeated. The extracts were combined.

For instrumental analysis, 5 μL of the fish extract or 60 μL of the water/methanol mixture (water samples) was separated on an Acquity UPLC BEH C18 column fitted with a BEH C18 pre-column and analyzed on a TSQ Quantiva triple quadrupole mass spectrometer. The mobile phase was methanol (10 mM ammonium acetate) and Milli-Q-filtered water (10 mM ammonium acetate) (see Table S5 for the liquid chromatography program). To avoid detergent blank peaks originating from cleaning mobile phase bottles, the methanol was prepared directly in the solvent bottle as delivered and Milli-Q was tapped into an empty methanol bottle. Furthermore, a guard column was fitted between the mobile phase and the autosampler to prevent background contamination from disturbing the analysis. All analytes were quantified against the BEHP internal standard, as it gave better reproducibility. The MS parameters are provided in Table S6. A 10-point calibration curve consisting of matrix-matched standards covering a concentration range of ∼3 orders of magnitude was used.

### Statistical Analysis

We assumed that the surfactant concentrations in fish over time can be described by a one-compartment fish model with first-order kinetics.^3^ The governing equation was
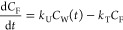
1where *C*_F_ is the surfactant concentration in fish (μg kg^–1^ ww), *t* is the time (h), *C*_W_(*t*) is the surfactant concentration in water (μg L^–1^) at time *t*, and *k*_U_ and *k*_T_ are the uptake and overall elimination rate constants (in units of L kg^–1^ h^–1^ and h^–1^, respectively). Table 2 lists the symbols used in the paper. The central goal of the analysis was to estimate *k*_U_ and *k*_T_ as well as the BCF (=*k*_U_/*k*_T_) from measurements of surfactant concentration in fish and water samples collected at different time points of the experiment. For the fish, only those time points were included for which *C*_F(measured)_ was above the limit of quantitation (LOQ) in all three fish (data below the LOQ will have a positive bias; including them would result in a negative bias in *k*_T_). In this analysis, there exist two sources of uncertainty: first, uncertainty in the actual concentration in water *C*_W_ at the time of sample collection and second, variability in the concentration in fish around the (expected) surfactant concentrations in fish assumed to follow model (1). We therefore set up a model in which we considered uncertainty in both water and fish measurements simultaneously by assuming a parametric model for the surfactant concentration in the water over time (either linear or quadratic) and accounted for the uncertainty in the corresponding parameters of the water model during the estimation of *k*_U_ and *k*_T_. This was done in a joint Bayesian model that we estimated via Hamiltonian Monte Carlo.

**Table 2:** Symbols Used in the Paper

symbol	explanation	unit
*A*	surface area of the epithelial membrane in the gills over which transport occurs	m^2^
*C*_F_	chemical concentration in fish	μg kg^–1^ ww^a^
*C*_W_	chemical concentration in water	μg L^–1^
*D*	diffusion coefficient of the chemical in the epithelial membrane in the gills	m h^–1^
*D*_FW_	fish–water equilibrium distribution ratio	L kg^–1^ ww
*D*_MLW_	membrane lipid–water distribution ratio	L kg^–1^
*f*_MLB_	mass-to-volume fraction of membrane lipid-like sorbent in the blood	kg L^–1^
*f*_MLF_	mass fraction of the fish that is membrane lipid or has equivalent partitioning properties	kg kg^–1^ ww
*k*_B_	elimination rate constant due to biotransformation	h^–1^
*k*_B-BCF_	*k*_B_ estimated from the *in vivo* BCF experiment	h^–1^
*k*_B-S9_	*k*_B_ estimated from the *in vitro* RT-S9 assay	h^–1^
*k*_U_	uptake rate constant	L kg^–1^ ww h^–1^
*k*_T_	overall elimination rate constant	h^–1^
*k*_2_	elimination rate constant due to gill respiration	h^–1^
*M*	mass of the fish	kg ww
ρ_M_	density of the epithelial membrane of the gills	kg L^–1^
*Q*_B_	rate of blood flow through the liver	L h^–1^
*Q*_LW_	clearance rate due to transformation of freely dissolved chemicals in the liver	L h^−1^
*t*	time	h
*V*_D_	volume of distribution	L

a Wet weight.

From the governing equation for the fish model (1), we can derive (see Text S2) the expected surfactant concentration in fish at a specific time point *t* as
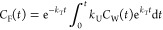
2This quantity depends on the surfactant concentration in the water over time, *C*_W_(*t*), that we modeled either based on a linear or a quadratic model, *C*_W_(*t*) = *b*_0_ + *b*_1_*t* or *C*_W_(*t*) = *b*_0_ + *b*_1_*t* + *b*_2_*t*^2^, respectively. It is then possible to solve the integral in (2) analytically and use the results for deriving the expected likelihood of the observed fish samples collected at different time points in terms of the parameters *k*_U_, *k*_T_, *b*_0_, *b*_1_, and *b*_2_ as well as two variance parameters for the measured concentrations in water and fish, respectively (see Text S2 for a detailed description of the model). We chose between the linear and quadratic model for the concentration in water in the first step by estimating a standard linear model with linear/quadratic time effect to the observed concentration data and selected the model with a lower Bayesian information criterion (BIC). In the full Bayesian model based on eq 2, we used uninformative priors for all parameters (independent of the first analysis step for the model of concentration in water) and monitored convergence and mixing of the chains based on trace plots and the R-hat statistic, as implemented in Stan.^22^ Uncertainty quantification for the model parameters and derived quantities was obtained from the posterior samples following standard practice in Bayesian inference. The analysis was performed in R^23^ using the cmdstanr package^24^ for implementation and estimation of the model. Text S3 describes how the uncertainty of several other derivative parameters was estimated and presents a simpler approach for estimating the BCF model based on a stepwise application of the analytical solution to eq 1 assuming constant *C*_W_ between water sampling time points. The results from this simpler approach were compared with the results of the Bayesian inference approach.

### *In Vitro* Estimation of Biotransformation

The *in vitro* intrinsic clearance by the S9 fraction of rainbow trout liver was measured for the same 11 anionic surfactants used in the BCF experiment as separate compounds. A mixture of 9 of these surfactants, without C_11_SO_3_ and C_13_SO_3_, was tested as well for a preliminary comparison of potential inhibition of transformation due to the presence of co-solutes. An established RT-S9 substrate depletion assay was employed,^25−27^ predating the currently available OECD guideline 319B.^28^ A single batch of the characterized liver homogenate material was prepared at the US EPA lab in Duluth and shipped on dry ice to the testing facilities at the University of Amsterdam. Properties and measured activity levels are presented in Table S7. The test was conducted using the same method applied for a series of cationic surfactants in Droge et al.^29^ and is summarized together with the *in vitro*–*in vivo* extrapolation in Text S4.

## Results and Discussion

### Quality Assurance of the Analytical Methods

For the water method, the repeatability, quantified as the average relative standard deviation of the 14 sets of triplicate samples collected during the exposure phase, ranged between 3 and 25% (Table S8). Repeatability was better for the shorter-chain compounds. Testing in a pre-experiment had shown better repeatability (generally <5%) in aquarium water, but the aquarium contained far fewer fish than during the BCF exposure experiment.

For fish analysis, method precision was assessed via triplicate injections of one extract (instrumental precision) and triplicate extraction of the same homogenate (extraction + instrumental precision). The instrumental precision ranged from 3 to 15%, and the extraction + instrumental precision ranged from 0.4 to 23%, whereby the longer-chain compounds showed better precision (Table S9). One reason for the poorer precision of the shorter-chain compounds, in particular, C_10_SO_3_ and C_11_SO_3_, could be the proximity of the concentrations to the LOQ. In general, the extraction + instrumental precision was poorer than the instrumental precision by itself, indicating that the extraction step made a significant contribution to the extraction + instrumental precision. DOSS was a notable exception, as it had the poorest instrumental precision (15%) and the best extraction + instrumental precision (0.4%). This is likely a reflection of the limited quantity of data for this assessment. The precision was judged to be satisfactory for direct injection of a complex matrix and sufficient for the purpose of this study.

The test chemical concentrations in the control fish and the fish method blanks were <1 ng g^–1^ ww in most cases. The LOQ of the method (calculated as mean + 10× standard deviation of the control fish) ranged from 0.2 to 8 ng g^–1^ ww (Table S10).

### Concentrations in Water

The surfactant concentrations in the aquarium water during the exposure phase showed a decreasing trend for some substances (Figure S1). Over the 4-day period, the concentrations decreased by a factor of ∼2 for C_13_SO_3_, C_14_SO_3_, and C_11_SO_4_ and a factor of 4 for C_13_SO_4_. The concentrations of these substances were relatively constant for the first 24 h. A possible explanation for this behavior is that a microbial population developed in the aquarium that biodegraded these substances.

Considering the decreasing time trend for some substances, the agreement with the nominal concentrations was evaluated using the data from the first 6 h of exposure. The relative standard deviation of the mean concentrations from the seven time points sampled ranged from 7 to 22% (Table 1). It was correlated with the precision of the analytical method (see Table S8), which suggests that the method uncertainty contributed significantly to the measured variability in the water concentrations. There was good agreement between the mean measured concentration and the nominal concentration for C_10_SO_3_, C_11_SO_3_, C_13_SO_3_, C_14_SO_3_, C_11_SO_4_, and C_10_-1-LAS (Table 1). The mean measured concentration was a factor of 1.7–2.0 higher than the nominal for C_13_SO_4_, C_12_-EO_4_-SO_4_, and DOSS. These were the analytes with the poorest precision in the analytical method. Although the method precision could not explain the discrepancy between the observed and nominal concentration, the correlation between precision and overestimation could be an indication that there were problems with the water method, for instance, during sampling, that led to a positive bias in the results. In contrast to these analytes, the mean measured concentration of C_16_SO_3_ was lower than the nominal concentration (a factor of 0.6). Given that C_16_SO_3_ was the most hydrophobic analyte, one possible explanation is sorption of C_16_SO_3_ to surfaces in the aquarium.

The concentration of all test chemicals was at least 100 times lower during the elimination phase than during the exposure phase (Table S11). The one exception was C_16_SO_3_, for which the concentration 1 h after the beginning of the elimination phase was just a factor of 30 lower, but 6 h later, the concentration had decreased to >100 times lower than at the end of the exposure phase.

In summary, two requirements for a BCF experiment were fulfilled, namely, measurable exposure and a strong gradient in exposure between the exposure phase and the elimination phase. The drift in water concentrations for some analytes shows that a dynamic kinetic model that accounts for changing exposure concentrations must be used to evaluate the data.

### Concentrations in Fish

All test chemicals were above the LOQ in the fish throughout most of the exposure phase except for C_10_SO_3_ (Figure S2). During the elimination phase, the concentrations of all test substances with alkyl chain lengths <14 eventually fell below the LOQ because of their rapid elimination.

For the experiment as a whole, the variability in concentrations between fish at a given time point ranged from 19 to 38% (mean RSD, Table S12). When a data point exceeded the mean of the concentration at the preceding and succeeding time points by >10 × mean RSD, it was treated as an outlier. This affected 19 of the ∼450 data points above the LOQ (Table S13). When they were removed, mean RSD decreased for several of the substances, and the range was lowered to 18–26% (Table S12). This was still considerably more than the repeatability of the analytical method (Table S9), indicating that there were significant interindividual differences in accumulation.

### Bioconcentration Factors (BCFs)

Semilogarithmic plots of concentration in fish during the elimination phase versus time yielded linear relationships for most test chemicals (Figure S2), indicating that elimination kinetics were first order and that a one-compartment fish model could be employed to estimate the BCF. The total elimination half-lives were <24 h for most of the test chemicals, which led to the concentrations of several falling below the LOQ before the end of the 7-day elimination phase. Sufficient data to quantify the elimination kinetics were obtained for all test chemicals except for C_10_SO_3_ and C_11_SO_3_, for which elimination was very rapid. BCF was estimated using the steady-state method for these two substances, whereby the plateau phase was assumed to extend between 6 and 96 h (Figure S2). The concentrations in fish during the plateau phase were both above and below the LOQ [but always above the LOD (mean + 3 × standard deviation of the control fish)]. To reduce the risk of introducing a positive bias into the BCF estimate, both the data above and below the LOQ were used. The Bayesian inference method as described in the Statistical Analysis section was employed to determine the BCF of the remaining test chemicals while accounting for the variability in *C*_W_. For C_16_SO_3_, there was a step increase in concentration in fish at the beginning of the accumulation phase, which indicates that there was a second, small, fast-responding compartment, perhaps due to sorption of the test chemical to the fish surface. Consequently, the data from the first 6 h were not included in the statistical analysis for C_16_SO_3_. The measured concentrations in water were used to evaluate the bioconcentration behavior. However, due to the possibility of a positive bias in the measured concentrations in water for some substances discussed above, we also provide BCFs calculated using the nominal concentrations in water for all test chemicals except C_16_SO_3_ (Table S14).

There was generally a good fit between the modeled and observed concentrations in fish (Figure S2) and the posterior distribution of the BCF estimates was quite narrow (Figure S3). The one-compartment fish model provided a good description of the uptake and elimination of all test chemicals. There was a pronounced response of the modeled concentration in fish to the decreasing concentrations in water during the second half of the exposure phase, confirming the need for a dynamic kinetic model to describe the experimental observations. The simpler model parameter estimation method using a stepwise application of the analytical solution to eq 1 provided values for BCF, *k*_U_, and *k*_T_ that were similar to those obtained with the Bayesian method (compare Tables 3 and S15), indicating that the simpler approach is also valid. Its major disadvantage was the less sophisticated evaluation of uncertainty, which resulted in broader 95% confidence intervals for the model parameters.

**Table 3:** Uptake Rate Constant (*k*_U_), Elimination Rate Constant (*k*_T_), and Bioconcentration Factor (BCF) of the Test Chemicals Determined from Measured Concentrations in Water, Together with the Membrane Lipid–Water Distribution Ratio (*D*_MLW_), the Diffusion Coefficient (*D*)^b^, Gill Elimination Rate Constant (*k*_2_),^b^ Biotransformation Rate Constant (*k*_B-BCF_)^b^ Estimated from the *In Vivo* Kinetic Data, and the Biotransformation Rate Constant Estimated from the *In Vitro* RT-S9 Assay (*k*_B-S9_)^a^^,^^b^

chemical	*k*_U_ (L kg^–1^ ww h^–1^)	*k*_T_ (h^–1^)	BCF (L kg^–1^ ww)	log *D*_MLW_ (L kg^–1^)	diffusion coefficient (*D*) (m h^–1^)	*k*_2_ (h^–1^)	*k*_B-BCF_ (h^–1^)	*k*_B-S9_ (h^–1^)
C_10_SO_3_	nq^c^	nq^c^	0.042^d^ (0.034–0.050)	3.01^e^	nq^c^	nq^c^	nq^c^	0.366 (0.33–0.40)
C_11_SO_3_	nq^c^	nq^c^	0.136^d^ (0.114–0.158)	3.39^g^	nq^c^	nq^c^	nq^c^	0.225 (0.21–0.24)
C_13_SO_3_	0.25 (0.22–0.29)	0.057 (0.052–0.062)	4.5 (4.1–5.0)	4.46^e^	3.5 × 10^–8^	0.00071	0.056 (0.051–0.061)	0.032 (0.028–0.036)
C_14_SO_3_	0.70 (0.61–0.80)	0.024 (0.022–0.026)	30 (26–33)	4.95^e^	3.1 × 10^–8^	0.00063	0.023 (0.021–0.025)	0.002^h^
C_16_SO_3_	6.3 (5.3–7.4)	0.0047 (0.0026–0.0062)	1370 (1070–2200)	6.19^g^	1.6 × 10^–8^	0.00033	0.0043 (0.0023–0.0059)	0.002^h^
C_11_SO_4_	0.136 (0.113–0.163)	0.178 (0.151–0.206)	0.77 (0.68–0.87)	4.16^g^	3.7 × 10^–8^	0.00076	0.177 (0.150–0.205)	0.151 (0.136–0.166)
C_13_SO_4_	1.62 (1.38–1.91)	0.048 (0.043–0.053)	34 (29–40)	5.21^e^	3.9 × 10^–8^	0.00080	0.047 (0.042–0.052)	0.028 (0.022–0.034)
C_10_-1-LAS	0.77 (0.67–0.87)	0.047 (0.044–0.050)	16.3 (14.6–18.2)	5.10^f^	2.4 × 10^–8^	0.00049	0.047 (0.044–0.050)	0.041 (0.037–0.045)
C_12_-EO_4_-SO_4_	0.090 (0.074–0.109)	0.100 (0.083–0.120)	0.89 (0.80–1.01)	4.24^f^	2.0 × 10^–8^	0.00041	0.100 (0.083–0.120)	0.084 (0.059–0.109)
DOSS	0.090 (0.074–0.110)	0.063 (0.056–0.072)	1.42 (1.22–1.66)	4.58^f^	9.3 × 10^–9^	0.00019	0.063 (0.056–0.072)	0.011 (0.009–0.013)

a The 95% credible/confidence interval is provided in brackets.

b Discussed in the sections Uptake Rate Constant and Elimination Rate Constant.

c Not quantifiable.

d Steady-state BCFs (as opposed to kinetic BCFs for the other test chemicals).

e Measurement from Droge.^18^

f Measurement from Droge et al.^19^

g Estimated using QSAR from Droge et al.^19^

h <Lower limit of the RT-S9 assay to detect clearance (LL_S9_), the value reported is LL_S9_/3.

The BCF values calculated from the measured concentrations in water ranged from 0.04 L kg^–1^ ww for C_10_SO_3_ to 1370 L kg^–1^ ww for C_16_SO_3_ (Table 3). This shows that it is possible to measure BCFs ranging over 4.5 orders of magnitude in one experiment. The proximity of the BCF for C_16_SO_3_ to the B threshold under REACH (2000 L kg^–1^)^30^ indicates that some anionic surfactants may be subject to classification as bioaccumulative.

Despite the limited high-quality measurements of the BCF of anionic surfactants in the literature, several comparisons with our data are possible. Our result for C_10_-1-LAS can be compared with the pioneering work of Johannes Tolls for C_11_-2-LAS, a molecule with a similar structure. Tolls’ measurements were also conducted in rainbow trout and at a similar water hardness.^4^ Our value for C_10_-1-LAS, 16.3 L kg^–1^, is similar to the reported value for C_11_-2-LAS, 6 L kg^–1^. It is also similar to the value of 17.2 L kg^–1^ determined in Senegalese sole.^8^ Our values for C_11_SO_4_ (0.77 L kg^–1^) and C_13_SO_4_ (34 L kg^–1^) bracket literature values for C_12_SO_4_ reported for several species (1–7.15 L kg^–1^),^1^ while our value for C_13_SO_4_ (34 L kg^–1^) is higher than a value for C_14_SO_4_ (11 L kg^–1^) measured in *Cyprinus carpio*.^11^ Our BCF for DOSS (1.42 L kg^–1^) compares with a literature value for rainbow trout carcass of 3.78 L kg^–1^.^31^ Note that the literature values for all substances except LASs, with possibly one exception, were determined by LSC of radiolabeled compounds and are thus upper boundaries for compound-specific BCFs.

**Figure 1:**
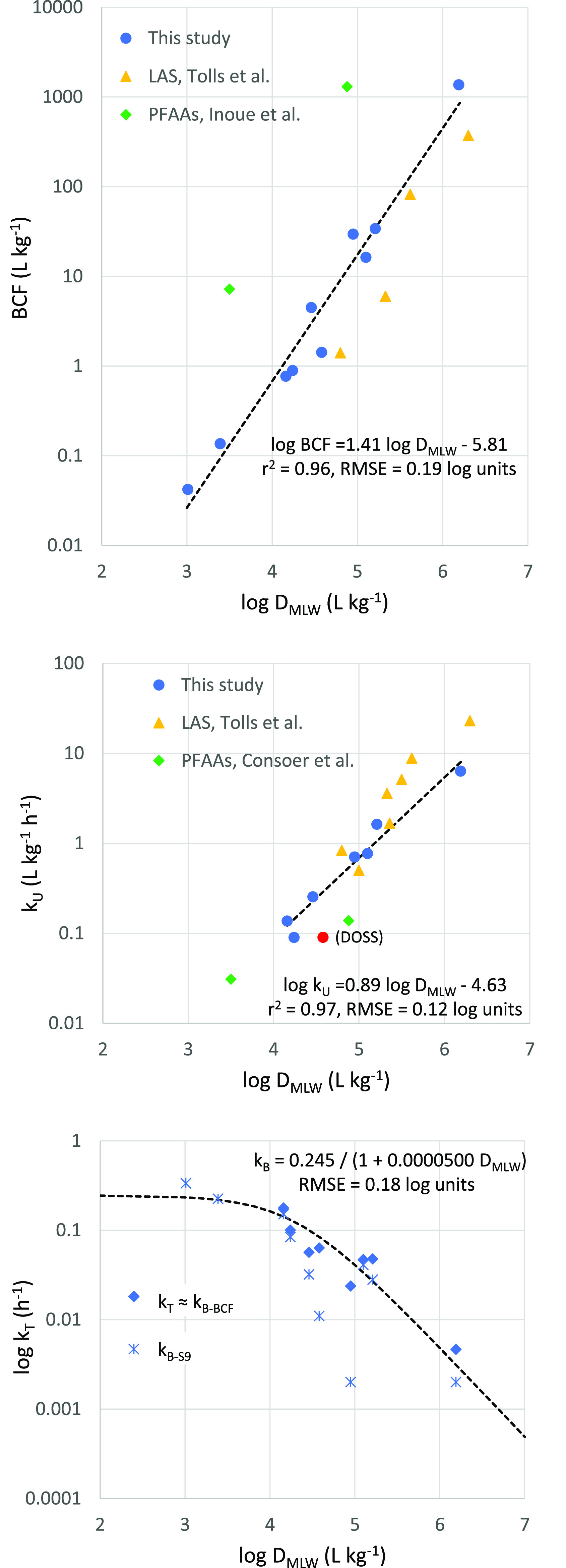
BCF and kinetic parameters from the bioconcentration experiment plotted against the log membrane lipid–water distribution ratio (*D*_MLW_). Upper panel: BCF; the line shows the linear regression of the two variables for the data from this study. Middle panel: Uptake rate constant (*k*_U_); the line shows the linear regression of the blue data points. The data from Consoer et al.^33,34^ were size-corrected to 24 g using the algorithm provided in their paper.^34^ Lower panel: Overall elimination rate constant (*k*_T_) and biotransformation rate constant determined using an *in vitro* RT-S9 assay (*k*_B-S9_); the line shows the model described in the text.

Anionic surfactants partition more than 100 000 times more strongly into membrane lipids than into neutral lipids.^19^ The membrane lipid–water distribution ratio (*D*_MLW_) is therefore expected to play a greater role in the bioconcentration of anionic surfactants than the neutral lipid–water distribution ratio (which is often approximated as the octanol–water distribution ratio *D*_OW_). Measured values of *D*_MLW_ have been reported for seven of the test chemicals (Table 3). For the remaining three test chemicals, *D*_MLW_ has been estimated using QSARs built from *D*_MLW_ measurements of structurally similar anionic surfactants.^19^ We found an excellent correlation between log BCF and log *D*_MLW_ (*r*^2^ = 0.96, Figure 1, upper panel). *D*_MLW_ had a very strong influence on BCF; an increase in *D*_MLW_ by a factor of 10 corresponded to an increase in BCF by a factor of 25. BCFs determined for 4 LAS isomers in rainbow trout held at 14 °C are also strongly correlated with *D*_MLW_ and lie close to the regression line obtained from our data.^4^ However, two persistent perfluorinated anionic surfactants, PFOA and PFOS,^32^ lay approximately two log units above the regression line (Figure 1, upper panel). To understand how *D*_MLW_ influences BCF, it is necessary to study the kinetic parameters for uptake and elimination.

### Uptake Rate Constant

The uptake rate constant, *k*_U_, ranged from 0.090 L kg^–1^ h^–1^ for DOSS to 6.3 L kg^–1^ h^–1^ for C_16_SO_3_ (it could not be measured for C_10_SO_3_ and C_11_SO_3_, Table 3). For all of the chemicals except C_16_SO_3_, *k*_U_ was markedly lower than the median *k*_U_ of 266 L kg^–1^ day^–1^ (11.1 L kg^–1^ h^–1^) that we measured for 10 neutral chemicals in somewhat larger rainbow trout.^35^ This indicates that the primary resistance for uptake does not lie in transport through water to the surface of the gill membranes, as in this case *k*_U_ would be similar for all of these compounds. Instead, transport through the membranes into blood must be rate limiting. This is consistent with the passive diffusion of small organic molecules through membranes being much slower for ions than for comparable neutral structures.^36^

Like the BCF, *k*_U_ was positively correlated with *D*_MLW_. When the bulky structure with two branched chains, DOSS, was excluded, then the slope of the regression of log *k*_U_ and log *D*_MLW_ became 0.89 with a 95% confidence interval that intersected 1 (Figure 1, middle panel). This means that the relationship between *k*_U_ and *D*_MLW_ was also approximately linear. *k*_U_ measured for seven LAS isomers in fathead minnows held at an average temperature of 21.4 °C show a similar dependence on *D*_MLW_ and lie close to the regression line determined from our data.^4^*k*_U_ reported for PFOA in rainbow trout in respirometer-metabolism chambers at 11 °C when size-corrected to 24 g fish agree well with the regression,^34^ while PFOS falls a factor 4 below the regression line (Figure 1, middle panel).^33^

Presuming that uptake occurs primarily via diffusion across the gills and that the primary resistance to diffusive mass transport is the epithelial membrane of the gills, then *k*_U_ (L kg^–1^ ww h^–1^) can be expressed as follows (derivation provided in Text S5):
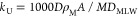
3where *D* is the diffusion coefficient of the chemical in the membrane (m h^–1^), ρ_M_ is the membrane density (kg L^–1^), *A* is the surface area over which diffusive transport occurs (m^2^), *M* is the fish mass (kg ww), and 1000 is a unit conversion factor (L m^–3^). Measured values of *D*_MLW_ were available (Table 3), *A* was estimated from the average fish weight (0.024 kg) according to the equation specific for estimating the gill surface area of rainbow trout from Hughes et al.^37^ corrected for the units used here, and ρ_M_ was set to 1 kg L^–1^, allowing eq 3 to be solved for *D*. While the magnitude of *D* is sensitive to errors in the estimates of ρ_M_, *A*, and *M*, the relative differences in *D* between chemicals are not affected by such errors. The calculated *D* varied between 9.3 × 10^–9^ and 3.9 × 10^–8^ m h^–1^ (Table 3), indicating that diffusive transport across the membrane is influenced by other molecular properties besides *D*_MLW_. The diffusion cross section of the molecule is one property that can be expected to affect *D*. With the exception of C_16_SO_3_, similar values of *D* were obtained for the sulfates and sulfonates, which are consistent with them having similar head groups and a single unbranched alkyl tail that would not impede passage into the membrane. Somewhat smaller *D* values were obtained for C_10_-1-LAS and C_12_-EO_4_-SO_4_, suggesting that the head groups of these molecules may have a somewhat larger diffusion cross section. A markedly smaller *D* value was obtained for DOSS (a factor of 4 less than the sulfates and sulfonates). DOSS has a dialkyl structure with branched chains, which one would expect to make the diffusion cross section larger.

In summary, the variability in *k*_U_ was primarily driven by the extent to which the chemical partitions from water into the gill membranes. For the bulkier substance DOSS, there was a substantial additional effect on the diffusion coefficient arising from its larger diffusion cross section.

### Elimination Rate Constant

The elimination rate constants *k*_T_ varied by a factor of 40, between 0.0047 and 0.178 h^–1^ (Table 3). There was a negative correlation between *k*_T_ and *D*_MLW_ (Figure 1, lower panel).

From eq 3 it follows that *k*_T_ can be defined as (derivation provided in Text S5)
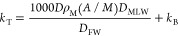
4where *D*_FW_ is the fish–water equilibrium distribution ratio (L kg^–1^) and *k*_B_ is the whole-body biotransformation rate constant (h^–1^). Membranes are an important tissue component for the storage of ionic surfactants in fish and a useful model for other tissue components that sorb amphiphilic chemicals.^19^ Therefore, *D*_FW_ was approximated as
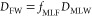
5where *f*_MLF_ is the mass fraction of the fish that is membrane lipid or has equivalent partitioning properties. Substituting this into eq 4 yields
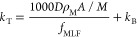
6The first term on the right-hand side of this equation is the rate constant for gill elimination *k*_2_. It was estimated using the estimates of the diffusion coefficient (*D*) from Table 3, the values of ρ_M_, *A*, and *M* given in the Uptake Rate Constant section, and assuming a value of 0.0125 for *f*_MLF_.^38,39^ Note that the latter accounts only for the contribution of phospholipids to the sorption capacity of the fish, thus possibly underestimating *f*_MLF_ and, consequently, resulting in an overestimation of *k*_2_. An estimate of *k*_B_ from the BCF experiment (*k*_B-BCF_) was then determined from the difference between measured *k*_T_ and the estimated *k*_2_.
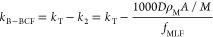
7*k*_B-BCF_ was greater than *k*_2_ for all chemicals (Table 3), indicating that biotransformation was the primary pathway of elimination. The difference was at least an order of magnitude, meaning that the estimated contribution of respiration to overall elimination was negligible.

Recently, the concept of a baseline screening BCF was introduced for surfactants, based on equilibrium partitioning assuming no biotransformation.^19^ The baseline screening BCF for the study chemicals exceeded the measured BCF by 1–2.5 orders of magnitude (Table S16), providing further evidence that biotransformation is an important factor influencing the elimination—and thereby the BCF—of the anionic surfactants studied here in rainbow trout. The deviation of PFOA and PFOS from the regression between BCF and *D*_MLW_ (Figure 1, upper panel) is attributable to them not being biotransformed.

*k*_B_ was also estimated from the RT-S9 assays conducted with individual chemicals (*k*_B-S9_). The RT-S9 assay yielded statistically significant clearance for eight of the test chemicals (Table S17 and Figure S4), while for two (C_14_SO_3_ and C_16_SO_3_), no significant clearance was measured and the lower limit of the RT-S9 assay to detect clearance (LL_S9_) was used to estimate *k*_B-S9_ (see Text S6). There was good agreement (within a factor of 3) between *k*_B-S9_ and *k*_B-BCF_ for six of the eight test chemicals for which *k*_B-BCF_ could be measured (Table 3). This result provides some confidence in the utility of the RT-S9 assay and the IVIVE estimation method. We note that the RT-S9 assay with the anionic surfactant mixture yielded significantly lower *k*_B-S9_ values compared to the single chemical RT-S9 assay for three out of six chemicals (Table S18, Figure S5, and Text S6). The mixture effect was observed for rapidly transformed shorter-chain analogues and thus mostly for structures with a lower BCF. The influence of co-solutes on the RT-S9 assay and how this might relate to *in vivo* biotransformation behavior deserve further investigation.^40^

The substances showing the poorest agreement between *k*_B-BCF_ and *k*_B-S9_ were C_14_SO_3_ and DOSS. In both cases, *k*_B-S9_ was less than *k*_B-BCF_ (by a factor of 11 and 6, respectively). Possible explanations for the discrepancy include an overestimation of *k*_T_, an under-estimation of *k*_2_, or an under-estimation of *k*_B-S9_. A significant overestimation of *k*_T_ can be ruled out for C_14_SO_3_, for which the 95% credible interval was 0.022–0.026 h^–1^ (see Table 3). For DOSS, on the other hand, there were few observations during the elimination phase of the experiment and there was considerable variability in the fish concentrations, leading to a broader 95% credible interval of 0.056–0.072 h^–1^ (see Table 3). A significant under-estimation of *k*_2_ is an unlikely explanation, as we are aware of no plausible hypotheses that could increase *k*_2_ to the extent that it would influence *k*_B-BCF_. An under-estimation of *k*_B-S9_ by up to a factor of ∼3 is possible for C_14_SO_3_, as this chemical was below the LL_S9_ and therefore LL_S9_/3 was used to estimate *k*_B-S9_. A factor of 3 would be sufficient to explain a significant part of the difference between *k*_B-BCF_ and *k*_B-S9_ for C_14_SO_3_. As reported in more detail in Text S6, the RT-S9 depletion assay performed with the mixture of anions indicated a depletion slope significantly different from 0 for both C_14_SO_3_ and C_16_SO_3_, though still just at the apparent LL_S9_. This would result in a higher k_B_ of a factor of 2.7 and 2.5, respectively, compared to the assumed LL_S9_/3 from the single-compound RT-S9 assay. Another factor that may explain lower *k*_B-S9_ compared to *k*_B-BCF_ is that the former only considers biotransformation in the liver, while extra-hepatic biotransformation may be a contributing factor for some chemicals. Identification and comparative study of the toxicokinetics of metabolites in the *in vivo* and *in vitro* systems could provide more insight into these questions.

The *k*_B-S9_ data are also plotted in Figure 1 (lower panel). In addition to visualizing the comparison above (since *k*_2_ is negligible, *k*_T_ in the figure is approximately equal to *k*_B-BCF_), they provide more insight into the influence of *D*_MLW_ on *k*_B_. In contrast to *k*_B-BCF_, *k*_B-S9_ was available for C_10_SO_3_ and C_11_SO_3_, which have lower *D*_MLW_ than the other test chemicals. The combined dataset suggests that *k*_B_ is approximately constant up until log *D*_MLW_ = 4 and thereafter transitions into a regime where *k*_B_ is inversely proportional to *D*_MLW_.

To explore the influence of *D*_MLW_ on *k*_B_, a simple mathematical model of anionic surfactant elimination via the liver was created. We assumed that the liver behaves like a well-mixed reactor and that the blood/water (dissolved phase) distribution ratio is proportional to *D*_MLW_ (see Text S7). This assumption is based on partitioning data for alkyl sulfates and sulfonates indicating a >100 000 times larger affinity for phospholipids compared to storage lipids (fish oil) and the expectation that *D*_MLW_ provides a relative indication of sorption to other amphiphilic blood components such as proteins for these anionic surfactants. The resulting equation for *k*_B_ was
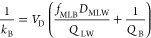
8where *V*_D_ is the volume of distribution (L), *f*_MLB_ is the mass-to-volume fraction of membrane lipid-like sorbents in the blood (kg L^–1^), *Q*_LW_ is the clearance rate due to transformation of freely dissolved chemicals in the liver (L h^–1^), and *Q*_B_ is the rate of blood flow through the liver (L h^–1^). This model fits the characteristics described above: at low *D*_MLW_, *k*_B_ is constant, and at high *D*_MLW_, *k*_B_ is inversely proportional to *D*_MLW_.

Assuming *V*_D_*f*_MLB_/*Q*_LW_ and *Q*_B_ to be constants, eq 8 was fitted to *D*_MLW_ and *k*_B_ for the 10 test chemicals (*k*_B-BCF_ for the eight chemicals for which it was available, *k*_B-S9_ for C_10_SO_3_ and C_11_SO_3_, Table 3), yielding
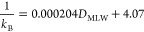
9with an RMSE = 0.18 log units (see Figure 1, lower panel).

Given that assuming *V*_D_*f*_MLB_/*Q*_LW_ to be constant gave a good fit to the data, and furthermore that *V*_D_ and *f*_MLB_ are expected to be similar for the test chemicals, it follows that *Q*_LW_, the clearance rate due to transformation of freely dissolved chemicals in the liver (L h^–1^), is also quite similar for the test chemicals. This is consistent with the observation that biotransformation of most anionic surfactants that possess linear alkyl chains occurs via a common mechanism involving omega-oxidation followed by β-oxidation.^20,41^ Our results suggest that it may be possible to predict the *k*_B_ of some anionic surfactants from *D*_MLW_ with good accuracy.

### Summary of the Chemical Properties Influencing BCF

The BCF of the anionic surfactants studied here is highly correlated with *D*_MLW_. *D*_MLW_ affects bioconcentration in two ways that amplify each other such that BCF increases more rapidly than *D*_MLW_. The first is the positive influence of *D*_MLW_ on the uptake rate constant. We attribute this to the greater partitioning from water into the gill membrane, which in turn amplifies the gradient for diffusive transport of the anionic surfactant across the membrane into the fish. The second is the negative influence of *D*_MLW_ on the elimination rate constant. We attribute this to the reduced availability of the surfactant for interaction with degrading enzymes because of sequestration of the surfactant from the freely dissolved form into the sorbed form. Since log *D*_MLW_ is positively correlated with alkyl chain length, the consequence is a pronounced increase in BCF with alkyl chain length.

Two other chemical properties also modulate the BCF. The membrane diffusion coefficient of the chemical decreases as its diffusion cross section gets larger, reducing passive transport across the gills and thereby the uptake rate constant. For the spectrum of chemicals studied here, this effect amounted to a factor of 4. The second modulating property is the inherent reaction rate of the freely dissolved chemical. This appeared to be similar for the surfactants studied.

More information is desirable for other classes of anionic surfactants. Considering the use of multiple types of phosphorus-based anionic surfactants produced in the EU in the range of 100–1000 tonnes year^–1^ and their apparently low overall biodegradability, particularly for single-chain alkylphosphate structures (Table S1), further research on *D*_MLW_ and RT-S9, and additional validation with limited *in vivo* BCF studies, would be relevant. We also note that per- and polyfluorinated surfactants behave differently than the alkyl surfactants studied here, particularly with respect to their persistence and other unique molecular interactions *in vivo*.^33,34^

The very strong influence of *D*_MLW_ on BCF and the proximity of the BCF for C_16_SO_3_ (log *D*_MLW_ = 6.2) to the threshold for B classification under REACH suggest that anionic surfactants with higher *D*_MLW_ may exceed this threshold even though they are readily biotransformed. Thus, B evaluation is a particularly relevant component of chemical safety assessment for these anionic surfactants. The work presented here, as synthesized in eqs 3, 6, and 9 including the estimates for *D* in Table 3, represents a significant step forward in our ability to assess the BCF of this important class of chemicals without having to resort to *in vivo* testing.

## Supporting Information Available

Text providing details of the methods and models used; tables of anionic surfactants, experimental materials, sampling schedule, analytical parameters, method performance indicators, test chemical concentrations in water and fish, BCF model parameters and S9 assay results; figures illustrating test chemical concentrations in water, fish and S9 assays with model fits (PDF)
