# Supplementary material for: In Vivo Bioconcentration of 10 Anionic Surfactants in Rainbow Trout Explained by In Vitro Data on Partitioning and S9 Clearance
Source: Environ Sci Technol. 2022 Apr 25;56(10):6305–14. doi: 10.1021/acs.est.1c05543 (PMC9118553; doi:10.1021/acs.est.1c05543)
Supplement: Supplementary file 1 — es1c05543_si_001.pdf [file es1c05543_si_001.pdf]

SUPPORTING INFORMATION
for the paper

In vivo bioconcentration of 10 anionic surfactants in rainbow trout explained by in vitro data on partitioning and S9 clearance

Anton Ribbenstedt[1], James M. Armitage[2], Felix Günther[3], Jon A. Arnot[4,5],

Steven T.J. Droge[6], Michael S. McLachlan[1*]

[1]Department of Environmental Science, Stockholm University, 106 91 Stockholm, Sweden

[2]AES Armitage Environmental Sciences, Inc., Ottawa, Ontario K1L 8C3, Canada

[3]Department of Mathematics, Stockholm University, 106 91 Stockholm, Sweden

[4]ARC Arnot Research and Consulting Inc., Toronto, Ontario M4M 1W4, Canada

[5]Department of Physical and Environmental Sciences, University of Toronto Scarborough, Toronto, Ontario M1C 1A4, Canada

[6]Institute for Biodiversity and Ecosystem Dynamics (IBED), University of Amsterdam, 1090 GE Amsterdam, The Netherlands

E-mail contact: michael.mclachlan@aces.su.se

Table of Contents

**Text**		
S1	Details of the bioconcentration experiment	S3
S2	Estimation of the BCF model in a Bayesian setting	S6
S3	Estimation of the BCF model using a simpler approach	S8
S4	Trout liver S9 parent depletion assay details and IVIVE	S10
S5	Elaboration of uptake and elimination kinetics	S14
S6	Details of the trout liver S9 assay results	S15
S7	Model of biotransformation of ionic surfactants in fish	S17
**Tables**		
S1	Anionic surfactants with tonnage >100/y in the EU	S19
S2	Test chemical standards	S21
S3	Solvents	S22
S4	Sampling schedule	S23
S5	Liquid chromatography program	S23
S6	MS/MS MRM parameters	S24
S7	Characterization of the liver S9 batch	S25
S8	Precision of the measurement of test chemical concentration in water	S25
S9	Precision of the measurement of test chemical concentration in fish	S26
S10	Limit of quantification for analysis of test chemical concentration in fish	S26
S11	Concentrations of test chemicals in water during the experiment	S27
S12	Variability of test substance concentration in fish samples at a given time point	S29
S13	Concentrations of test chemicals in fish during the experiment	S30
S14	Uptake rate constant, elimination rate constant and bioconcentration factor of the test chemicals determined using nominal concentrations in water	S34
S15	Uptake rate constant, elimination rate constant and bioconcentration factor of the test chemicals determined from measured C_W using the simpler estimation method	S34
S16	Comparison of measured BCF and baseline BCF	S35
S17	S9 results for chemicals tested individually	S36
S18	S9 results for chemicals tested in a mixture	S37
**Figures**		
S1	Test chemical concentrations in water vs. time during the exposure phase	S38
S2	Test chemical concentrations in fish vs. time including fitted model	S40
S3	Statistical distribution of the estimate of BCF	S45
S4	RT-S9 depletion data for C11SO3 and C13SO3 obtained in experiments with individual compounds	S46
S5	RT-S9 depletion data for anionic surfactants tested in a mixture	S46

Text S1: Details of the bioconcentration experiment

Juvenile rainbow trout were purchased from Nordic trout Sweden AB, and held in the aquaria facility for one month prior to starting the experiment on October 3, 2019. The experiment was conducted in three 350 L fiberglass aquaria with a water renewal rate of ~1.5 L min^{-1} corresponding to a turnover time of ~3.3 h. The water temperature and the air temperature in the aquaria room were 10 °C. The lighting was dim and programmed on a 12 h light / 12 h dark cycle.

Three aquaria were used: one for the control fish, one for the exposure phase, and one for the elimination phase. Each aquarium was equipped with external circulating pumps (Eheim model 2273 Prof 4) containing a filter of polyester wool wadding. Aquarium water was pumped at 800-1200 L h^{-1} and the water discharge hose was placed on the screen covering the aquarium so that the resulting bubble entrainment provided for aeration. Each pump was equipped with a pre-filter housing (Eheim) that was filled with the same polyester wool. The main filter wadding was changed immediately prior to starting the experiment while the pre-filter wadding was exchanged daily. To further reduce the levels of organic material in the aquaria, the fish feces were siphoned off of the bottom of the aquaria each day, immediately before feeding and 1 h after the fish were fed.

The fish were fed once daily using fish pellets supplied by the fish farm at ~0.7 % of their body weight per day. Of the ~130 fish used in the experiment, no injuries, abnormal behaviour or deaths were observed.

For the exposure phase, a methanol solution of the test chemical mixture was infused continuously at 8 µL min^{-1} into the water inflow of the aquarium using a syringe pump. The nominal concentrations of the chemicals in water ranged from 5.6-52 µg L^{-1} (Table 1). They were selected to be as low as possible to minimize the risk of toxic effects while still being high enough to enable collection of high-quality data, building on the results of a preliminary exposure experiment. The syringe pump was started in an aquarium containing no fish. After 16 h to allow the concentrations to stabilize, ~100 rainbow trout were transferred to the aquarium. Over the following 4 d water samples were collected and fish were sacrificed according to the schedule in Table S4. The remaining fish were then transferred to a second aquarium that received water without chemical addition. Over the following 7 d further water samples were collected and fish were sacrificed. Fish were also sampled on days 1 and 4 from the control aquarium.

Water samples were collected at eight time points during the exposure phase and two time points during the elimination phase for the determination of TOC and alkalinity. TOC was measured with NDIR detection after high-temperature catalytic combustion. The pH ranged from 7.65 to 7.95 and the alkalinity averaged 1.178 mmol L^{-1} during the experiment (Table A below). The approximate carbonate hardness was 118 mg $CaCO_3$ L^{-1}. The TOC content of the water supply to the aquarium water was 3.89 mg C L^{-1}, but during the exposure phase the TOC concentration was 5.74±0.93 mg C L^{-1} (Table B). This indicates that the experimental conditions increased the TOC level by 2 mg C L^{-1}. Of this, the methanol from the test chemical infusion was estimated to account for 1.6 mg C L^{-1}. The large contribution of methanol was corroborated by the observation of lower TOC levels in the elimination aquarium (4.23±0.65 mg C L^{-1}) and the control aquarium (4.85±0.78 mg C L^{-1}). The results show that excretion of organic material by the fish contributed little to the TOC level in the aquaria.

Table A: Alkalinity and pH during the experiment

**Day**	**pH**	**Alkalinity (mmol L^{-1})**
No fish		
0	7.99	1.2684
Control aquarium		
1	7.8	1.2226
2	7.8	1.2096
3	7.76	1.1504
4	7.79	1.1907
5	7.73	1.1443
6	7.79	1.1432
7	7.84	1.1466
8	7.84	1.1836
Test aquaria		
1	7.65	1.2313
2	7.68	1.2188
3	7.71	1.1551
4	7.77	1.1941
5	7.76	1.1475
6	7.89	1.1412
7	7.9	1.1485
8	7.95	1.1866

Table B: TOC during the experiment

Day	Hour	TOC (mg C L^{-1})
No fish		
0		3.9
Control aquarium		
1	0	5.1
1	1	6.5
1	6	4.2
2	24	5.0
3	48	4.4
4	72	5.3
5	96	4.0
5	106	4.2
8	168	4.9
Test aquaria - Exposure		
1	0	6.9
1	1	3.9
1	6	5.8
2	24	6.1
3	48	6.0
4	72	5.4
5	96	6.1
Test aquaria - Elimination		
5	106	3.8
8	168	4.7

Text S2: **Estimation of the BCF model in a Bayesian setting**

<u>Derivation of the formulas</u>

Based on the model (1) from the main text

$$\frac{dC_F}{dt} = k_U C_W(t) - k_T C_F$$

we derive the fish concentration C_F at a specific time t. To solve this linear differential equation, we proceeded as follows:

1. Put differential equation in standard form:

$$\frac{dC_F}{dt} + p(t)C_F = f(t)$$
$$\frac{dC_F}{dt} + k_T C_F = k_U C_W(t)$$

2. Find the integrating factor:

$$\mu(t) = e^{\int p(t)dt} = e^{\int k_T dt} = e^{k_T t}$$

3. Multiply the DE by the integrating factor

$$e^{k_T t}\frac{dC_F}{dt} + e^{k_T t}k_T C_F = k_U C_W(t)e^{k_T t}$$

4. Corresponds to: $\frac{d}{dt}(e^{k_T t}C_F) = k_U C_W(t)e^{k_T t}$
5. Integrate both sides by t:

$$e^{k_T t}\, C_F(t) = \int_0^t k_U C_W(t)e^{k_T t}\, dt$$

6. Solve for the fish concentration at time t:

$$C_F(t) = e^{-k_T t}k_U \int_0^t C_W(t)e^{k_T t}dt. \qquad (I)$$

The integral in equation (I) depends on the assumed (parametric) model for the water concentration over time, $C_W(t)$. In case of the linear model $C_W(t) = b_0 + b_1 t$, we can derive

$$\begin{aligned} C_F(t) &= e^{-k_T t}k_U \int_0^t (b_0 + b_1 t)e^{k_T t}dt \\ &= e^{-k_T t}k_U \left(b_0 \int_0^t e^{k_T t}dt + b_1 \int_0^t t\, e^{k_T t}dt\right) \\ &= e^{-k_T t}k_U \left(b_0 \frac{e^{k_T t} - 1}{k_T} + b_1 \frac{e^{k_T t}(k_T t - 1) + 1}{k_T^2}\right) \qquad (II) \end{aligned}$$

In case of the quadratic model $C_W(t) = b_0 + b_1 t + b_2 t^2$:

$$C_F(t) = e^{-k_T t} k_U \int_0^t (b_0 + b_1 t + b_2 t^2) e^{k_T t} dt$$
$$= e^{-k_T t} k_U \left(b_0 \int_0^t e^{k_T t} dt + b_1 \int_0^t t\, e^{k_T t} dt + b_2 \int_0^t t^2\, e^{k_T t} dt \right)$$
$$= e^{-k_T t} k_U \left(b_0 \frac{e^{k_T t} - 1}{k_T} + b_1 \frac{e^{k_T t}(k_T t - 1) + 1}{k_T^2} + b_2 \frac{e^{k_T t}(k_T t(k_T t - 2) + 2) - 2}{k_T^3} \right) \quad (III)$$

Specification of the Bayesian model in Stan

Assume we have measurements W of the surfactant concentration in N_W water samples collected at time points T_W as well as measurements F on the surfactant concentration in N_F fish collected at time points T_F.

In our Bayesian model, we assumed that the water measurements W are Gaussian distributed with expectation depending on the time point of sample collection T_W in a linear or quadratic fashion and standard deviation σ_W:

$$W | \mathrm{T_W}, b_0, b_1, \sigma_W \sim Normal(b_0 + b_1 \mathrm{T_W}, \sigma_W),$$

or

$$W | \mathrm{T_W}, b_0, b_1, b_2, \sigma_W \sim Normal(b_0 + b_1 \mathrm{T_W} + b_2 T_W^2, \sigma_W).$$

For the measurements in fish we assume a log-Normal distribution that accounts for increased variance in measurements of high surfactant concentration among the fish. The expectation of the log-Normal corresponds to $log\big(C_F(T_F)\big)$ as derived from equation (II) or (III) in supplement S2.

$$F | T_F, b_0, b_1, b_2, k_T, k_{U,} \sigma_F \sim LogNormal\big(log\big(C_F(T_F)\big), \sigma_F\big).$$

We estimate the set of parameters $(b_0, b_1, b_2, k_T, k_{U,} \sigma_F, \sigma_W)$ in a joint MCMC-based analysis. The parameters $(b_0, b_1, b_2, \sigma_W)$ are thereby mostly identified through the water measurements W, and $k_T, k_{U,} \sigma_F$ are identified through the fish measurements F. Due to the structural assumptions on the fish and water models necessary for modelling, there might however be some mutual influence between the two datasets and parameters of the fish/water models. This means that, e.g., the parameters estimated in the water model b_0, b_1, b_2 might be slightly different when fitting the model to the water observations separately compared to estimation in the joint analysis of fish and water data.

Text S3: Estimation of the BCF model using a simpler approach

In order to account for the observed variability in C_W (see below), the analytical solution to equation (1) in **Text S2** was applied in a stepwise from one sampling time point to the next, integrating over a time interval Δt equal to the time between the two sampling points. During each step, C_W was assumed to be constant and equal to the average of the concentrations measured at the start and the end of the time interval. The concentration in fish at the end of the interval $C_{F(t+\Delta t)}$ could be then calculated from the concentration at the beginning of the interval $C_{F(t)}$ by integrating equation (1) (**Text S2**) over the time interval:

$$C_{F(t+\Delta t)} = \left(C_{F(t)} - \frac{k_U}{k_T} C_W\right) e^{-k_T \Delta t} + \frac{k_U}{k_T} C_W \qquad (2)$$

k_U and k_T were fitted simultaneously for the complete experiment (uptake phase and depuration phase) using the solver function in Microsoft Excel. The fitting criterion was to minimize the sum of the normalized residuals ($C_{F(measured)} - C_{F(modeled)}$), whereby the normalization was done to the smaller of the measured and modeled concentrations:

$$fitting\ criterion = minimize\ \sum \frac{\left|\left(C_{F(measured)} - C_{F(modeled)}\right)\right|}{\mathrm{MIN}\left(C_{F(measured)}, C_{F(modeled)}\right)} \qquad (3)$$

Employing residuals as a fitting criterion introduces a strong bias towards data points with higher concentrations because the magnitude of the residual is greater at higher concentration for an identical percent deviation between observation and model. By normalizing the residuals, the bias associated with the magnitude of the concentration is eliminated. The fitting was done using $C_{F(measured)}$ for individual fish. Only those time points were included for which $C_{F(measured)}$ was above the LOQ in all three fish. BCF was calculated as the quotient of k_U and k_T.

This method for estimating the BCF model parameters while accounting for the variability of the concentration in water did not lend itself to the determination of model parameter uncertainty. We therefore estimated the model parameters with a standard procedure that provides parameter uncertainty estimates. The standard procedure assumes a constant concentration in water, and we assumed that the uncertainty in the standard procedure would be the same as the uncertainty in our procedure. Since our procedure was designed to give a better fit of the observations, this assumption is likely conservative, i.e., we would expect the better fitted model to have a somewhat lower uncertainty.

The fitting of the model while assuming constant C_W was conducted using the sequential method described in the OECD 305 guideline. First k_T was calculated from the depuration phase data assuming that C_W was negligible using a linear regression of ln C_F vs. time. Then k_U was calculated from the exposure phase data using a linear regression of C_F versus $C_W(1-e^{-k_T t})/k_T$, where C_W was the average concentration in water during the exposure phase. The standard error of the slopes of the regressions (k_T and k_U) were extracted using the LINEST function in excel. Error propagation was used to calculate the standard error in BCF. The relative standard errors of k_U, k_T and BCF were calculated as the measure of uncertainty for our best estimates of these model parameters.

k_U and k_T could not be determined for $C_{10}SO_3$ and $C_{11}SO_3$ because C_F was below the LOQ throughout the depuration phase. Consequently, BCF for these chemicals was determined using the steady state method. The uncertainty in BCF was estimated from the standard deviations of the C_F and C_W values used to calculate BCF.

Regarding the other derivative model parameters, the relative standard error of $k_{B\text{-}BCF}$ was assumed equal to that of k_T because k_2 made a negligible contribution to k_T for all substances. At a first approximation, the uncertainty in D can be approximated by the uncertainty in k_U. However, this does not account for the uncertainty in D_{MLW}, which applies not only to its measurement but also to its suitability as a model for the partitioning properties of the membranes in the gill epithelium.

Text S4: Trout liver S9 parent depletion assay details and IVIVE

Liver tissue was separately pooled from the same five rainbow trout (three males/two females, ~300 g, liver weight ~3 g) used also for the RT-S9 study on a series of cationic surfactants (Droge et al., 2021b). The liver sample was homogenized in an appropriate buffer and then centrifuged to yield a 13,000 x *g* supernatant (the 'S9 fraction', lacking only mitochondrial fractions). No further refinement towards microsomal fractions was made to remove cytosolic and lysosomal components. Ethoxyresorufin-O-deethylase (EROD, phase I dealkylation), UGT (phase II glucuronidation) and glutathione-S-transferase (GST) activity are 3.9 ± 0.2, 1179 ± 6, and 501 ± 18 pmol /min · mg protein, respectively. The RT-S9 content for this batch liver homogenate used was 2.16 mL RT-S9/g liver, and the RT-S9 fraction contained 23.8 mg protein/mL RT-S9 (micro Lowry assay). Upon arrival at the University of Amsterdam the liver S9 material was stored in a -80 ^{o}C freezer until use.

Individual chemicals were first dissolved in methanol and then further diluted to 20% methanol in 100 mM phosphate buffer to make a ~20 µM stock solution, which was then spiked as 10 µL in 190 µL of active S9 mixture. The active S9 mixture consisted of 100 mM potassium phosphate-buffered saline (PPBS) adjusted to pH 7.8, 2 mM β-NADPH, 2 mM UDPGA, 0.1 mM PAPS, 5 mM GSH, 10 µg/mL alamethicin (in methanol carrier; 0.1% v/v final concentration), and 2 mg/mL S9 protein. The substrate depletion assays were performed using the multiple vial method described in OECD TG 319B, employing two or three separately spiked series. Reactions were performed at 11 °C in a temperature-controlled water bath (B. Braun Frigomix-R) that was placed on a 2 cm travel path shaker (Gerhardt LS 10) set at 45 rpm. As a positive control alongside each active RT-S9 assay, a duplicate sample of *N*,*N*-dimethyl-dodecylamine was included to confirm depletion of >90% of parent compound within 60 minutes. Since Chen *et al*. (2014) found no other losses with inactivated RT-S9 for homologue alkylsulfates and alkylsulfonates, no additional assays with inactivated RT-S9 were performed.

Reactions were quenched with 600 µL ice-cold acetonitrile. After 10 s vortexing and 5 min centrifugation (Hermle Z300, 2500 rpm), 100 µL clear supernatant was transferred into 300 µL glass inserts placed in new autosampler vials, and mixed with 100 µL pure water. Concentrations were measured using a Prominence UFLC-XR system (Shimadzu) coupled to a tandem mass spectrometer (QTRAP 4000, Applied Biosystems) with with a Turbo Ion spray source operated at 400 °C. For chromatographic separations, a 100 x 2.1mm x 2.7 µm Supelco Ascentis Express column was used (40 ^{o}C), with eluent A: pure water with 2 mM ammonium acetate, and eluent B: methanol, following a gradient elution ramping up between 30-95%. External standards were prepared in 50% acetonitrile/water, calibration curves used covered at least four points in a log-linear range. The lowest external standard to fit the log-linear calibration curve served as limit of quantification, requiring a peak of at least 10x the average noise.

Each vial was treated as an independent vial with recorded exposure time. Log-transformed concentration data of the parent compound were plotted against exposure time (min) and analysed using Graphpad PRISM 8. When the slope was significantly different from 0 ($p<0.05$), the slope (min^{-1}) was multiplied by -2.3 and by 60 (min h^{-1}) to obtain a first order depletion rate constant (k_{dep}; h^{-1}).

Extrapolation of k_{dep} to whole body rate biotransformation rate constant ($k_{B\text{-}S9}$)

The basic approach to extrapolate first order depletion rate constants (k_{dep}) to whole body biotransformation rate constants (k_B) is well established in the literature (e.g., Nichols et al., 2013; Krause and Goss, 2018). The main steps of the in vitro to in vivo extrapolation (IVIVE)

are to i) convert k_{dep} to *in vitro* intrinsic clearance ($CL_{int,S9}$ mL h^{-1} mg S9 $protein^{-1}$), ii) convert *in vitro* intrinsic clearance to *in vivo* intrinsic clearance ($CL_{int,in\ vivo}$; mL h^{-1} g $liver^{-1}$), iii) convert *in vivo* intrinsic clearance to hepatic clearance (CL_B; mL h^{-1} kg bw^{-1}) and iv) convert hepatic clearance to whole-body biotransformation rate constant (k_B; h^{-1}). Hepatic clearance (CL_B) accounts for blood flow to the liver as the rate-limiting process and the conversion to k_B accounts for the internal distribution of the chemical in the organism.

The *in vitro* intrinsic clearance ($CL_{int,S9}$ mL h^{-1} mg S9 $protein^{-1}$) is calculated by dividing the k_{dep} (h^{-1}) by the concentration of S9 protein in the assay (2 mg S9/ml assay). The *in vivo* intrinsic clearance ($CL_{int,in\ vivo}$; mL h^{-1} g $liver^{-1}$) is calculated by multiplying the *in vitro* intrinsic clearance by an estimate of the amount of S9 protein per gram liver (147.8 mg g^{-1} liver). The *in vitro* and *in vivo* intrinsic clearances calculated from the depletion studies for the various anionic surfactants considered here are summarized in Table S17 and Table S18.

Hepatic clearance (CL_B; mL h^{-1} kg bw^{-1}) is calculated using the expression proposed by Krause and Goss (2018) shown below.

$$CL_B = \frac{Q_B \cdot CL_{in\ vivo,int} \cdot \frac{D_{Assay,W}}{D_{Blood,W}}}{Q_B + CL_{in\ vivo,int} \cdot \frac{D_{Assay,W}}{D_{Blood,W}}}$$

where Q_B is the blood flow to the liver (mL h^{-1} kg bw^{-1}), $CL_{in\ vivo,int}$ is the *in vivo* intrinsic clearance normalized to the whole body (i.e., now in units of mL h^{-1} kg bw^{-1}) and $D_{Assay,W}$ and $D_{Blood,W}$ are distribution ratios for the assay and blood vs. water. The *in vivo* intrinsic clearance normalized to the whole body is calculated by using an assumed liver fraction of whole body (0.015 kg liver/kg whole body).

The extrapolated whole body biotransformation rate constant ($k_{B\text{-}S9}$; h^{-1}) is calculated by dividing the hepatic clearance by the apparent volume of distribution reference to blood (VD_{SS}), i.e.,

$$k_{B-S9} = \frac{CL_B}{VD_{SS}}$$

where CL_B is the hepatic clearance of blood via biotransformation (converted to units of L h^{-1} kg bw^{-1}) and VD_{SS} is the apparent volume of distribution referenced to blood (L kg^{-1}).

Blood flow to the liver (Q_B; mL h^{-1} kg bw^{-1}) is calculated from the estimated total cardiac output (Q_T; mL h^{-1} kg bw^{-1}) and the estimated fraction flowing to the liver (0.259). Following Nichols et al. (2013), Q_T is estimated as a function of temperature (°C) and fish size (in g).

$$Q_B = 0.259 Q_T$$

$$Q_T = \left[(0.23T - 0.78) \cdot \left(\frac{BW}{500}\right)^{-0.1}\right] \cdot 1000$$

Assay-water and blood-water distribution ratios ($D_{Assay,W}$, $D_{Blood,W}$) are calculated from partitioning properties and estimates of the proximate composition of the assay medium and blood with respect to lipids, proteins and water.

$$D_{Assay,W} = 0.0005 \cdot D_{MW} + 0.002 \cdot D_{PW} + 0.9975$$

where 0.0005 is an estimate of the phospholipid content of the assay medium, 0.002 is the S9 protein content (from 2 mg/ml) and 0.9975 is the water content of the assay medium. D_{MW} is the membrane-water distribution ratio; D_{PW} is the protein-water distribution ratio

$$D_{Blood,W} = 0.007 \cdot D_{SlW} + 0.007 \cdot D_{MW} + 0.0225 \cdot D_{SaW} + 0.1235 \cdot D_{PW} + 0.84$$

where 0.007 is an estimate of the storage lipid content of whole blood, 0.007 is an estimate of the phospholipid content of whole blood, 0.0225 is an estimate of the plasma protein (serum albumin) content of whole blood, 0.1235 is an estimate of other proteins and 0.84 is the water content of whole blood. Accordingly, blood has a total lipid and organic matter content of 0.014 and 0.16 respectively, consistent with previous approaches to estimate blood-water partitioning. D_{SLW} is the storage lipid-water distribution ratio, D_{SaW} is the serum albumin-water distribution ratio.

The apparent volume of distribution referenced to blood (VD_{SS}; L kg^{-1}) is calculated following Nichols et al. (2013), i.e.,

$$VD_{SS} = \frac{D_{Fish,W}}{D_{Blood,W}}$$

$$D_{Fish,W} = 0.0375 \cdot D_{SlW} + 0.0125 \cdot D_{MW} + 0.003 \cdot D_{SaW} + 0.15 \cdot D_{PW} + 0.797$$

where 0.0375 is an estimate of the storage lipid content of the fish, 0.0125 is an estimate of the phospholipid content of the fish, 0.0225 is an estimate of the plasma protein (serum albumin) content of the fish (whole body level), 0.15 is an estimate of other proteins and 0.797 is the water content.

Measured (log D_{MW}) and estimated partitioning properties for the anionic surfactants considered here are as follows:

Chemical	log D_{SlW}	log D_{MW}*	log D_{SaW}	D_{PW}
$C_{10}SO_3$	1.74	3.01	2.86	0.09
$C_{11}SO_3$	2.12	3.39	3.12	0.39
$C_{13}SO_3$	3.18	4.46	3.88	1.24
$C_{14}SO_3$	3.66	4.95	4.22	1.63
$C_{16}SO_3$	4.89	6.19	5.09	2.61
$C_{11}SO_4$	2.88	4.16	3.67	1.00
$C_{13}SO_4$	3.92	5.21	4.40	1.84
C_{10}-1-LAS	3.81	5.10	4.33	1.75
C_{12}-EO_4-SO_4	2.96	4.24	3.72	1.07
DOSS	3.30	4.58	3.96	1.34
BEHP	2.53	3.81	3.42	0.73

* = measured; otherwise estimated based on SPLFERs (Endo et al., 2011ab; Endo et al. 2012) and scaling factors (Armitage et al. 2013).

<u>Other assumptions:</u>

Water temperature = 10°C

Fish body weight = 24g
Biological/blood/assay pH = 7.8

References

Armitage, J.M.; Arnot, J.A.; Wania, F.; Mackay, D. Development and evaluation of a mechanistic bioconcentration model for ionogenic organic chemicals in fish. *Environ. Toxicol. Chem.* **2013**, *32(1)*, 115-128.

Chen, Y.; Hermens, J.L.M.; Jonker, M.T.O.; Armitage, J.M.; Arnot, J.A.; Nichols, J.W.; Fay, K.A.; Droge, S.T.J. Which molecular features affect the intrinsic hepatic clearance rate of ionizable organic chemicals in fish? *Environ. Sci. Technol.* **2016,** *50*, 12722-12731.

Droge, S. T. J.; Armitage, J. M.; Arnot, J. A.; Fitzsimmons, P. N.; Nichols, J. W. Biotransformation potential of cationic surfactants in fish assessed with rainbow trout liver S9 fractions. *Environ. Chem. Toxicol*. **2021,** *40(11),* 3123-3136.

Endo, S.; Escher, B.I.; Goss, K-U. Capacities of membrane lipids to accumulate neutral organic chemicals. *Environ. Sci. Technol.* **2011a,** *45(14),* 5912-5921.

Endo, S.; Goss, K-U. Serum Albumin Binding of Structurally Diverse Neutral Organic Compounds: Data and Models. *Chem. Res. Toxicol*. **2011b**, *24(12)*, 2293–2301.

Endo, S.; Bauerfeind, J.; Goss, K-U. Partitioning of neutral organic compounds to structural proteins. *Environ. Sci. Technol.* **2012,** *46(22),* 12697-12703.

Krause, S.; Goss, K-U. In Vitro- in Vivo Extrapolation of Hepatic Metabolism for Different Scenarios - a Toolbox. *Chem. Res. Toxicol.* **2018**, *31(11)*, 1195-1202.

Nichols, J. W.; Huggett, D.B.; Arnot, J.A.; Fitzsimmons, P.N.; Cowan-Ellsberry, C.E. Toward improved models for predicting bioconcentration of well-metabolized compounds by rainbow trout using measured rates of in vitro intrinsic clearance. *Environ. Toxicol. Chem.* **2013**, *32(7)*, 1611-1622.

Text S5: Elaboration of uptake and elimination kinetics

According to the one compartment kinetic model with passive diffusive uptake of chemical, chemical exchange between the water and the fish can be described by the following equation if elimination due to fecal excretion and fish growth are negligible:

$$M \cdot \frac{dC_F}{dt} = 1000 \cdot k \cdot A \left(C_W - \frac{C_F}{D_{FW}} \right) - M \cdot k_B \cdot C_F \qquad (1)$$

where M is fish mass (kg), C_F is chemical concentration in fish (μg kg^{-1}), t is time (h), k is the mass transfer coefficient for diffusive transport between fish and water (m h^{-1}), A is the surface area over which this transport occurs (m^2), C_W is the chemical concentration in water (μg L^{-1}), D_{FW} is the fish-water equilibrium distribution ratio (L kg^{-1}), k_B (h^{-1}) is the rate constant for biotransformation, and 1000 is a conversion factor (L m^{-3}). Here we presume that uptake occurs primarily across the gills and that the primary resistance to diffusive mass transport is the epithelial membrane of the gills. In this case the mass transfer coefficient k can be expressed as the product of a diffusion coefficient in the membrane D (m h^{-1}), the membrane-water distribution ratio D_{MLW} (L kg^{-1}), and the membrane density ρ_M (kg L^{-1}) (Barber, 2003).

$$M \cdot \frac{dC_F}{dt} = 1000 \cdot D \cdot \rho_M \cdot A \cdot D_{MLW} \left(C_W - \frac{C_F}{D_{FW}} \right) - M \cdot k_B \cdot C_F \qquad (2)$$

Rearranging:

$$\frac{dC_F}{dt} = 1000 \cdot D \cdot \rho_M \cdot {}^{A}\!/_{M} \cdot D_{MLW} \cdot C_W - \left(\frac{1000 \cdot D \cdot \rho_M \cdot ({}^{A}\!/_{M}) \cdot D_{MLW}}{D_{FW}} + k_B \right) C_F \qquad (3)$$

Comparing this with the equation for the kinetic model used to evaluate the experimental data:

$$\frac{dC_F}{dt} = k_U \cdot C_W - k_T \cdot C_F \qquad (4)$$

yields:

$$k_U = 1000 \cdot D \cdot \rho_M \cdot {}^{A}\!/_{M} \cdot D_{MLW} \qquad (5)$$

and

$$k_T = \frac{1000 \cdot D \cdot \rho_M \cdot ({}^{A}\!/_{M}) \cdot D_{MLW}}{D_{FW}} + k_B \qquad (6)$$

<u>Reference</u>

Barber, M. C. A review and comparison of models for predicting dynamic chemical bioconcentration in fish. *Environ. Toxicol. Chem.* **2003**, *22*, 1963-1992.

Text S6: Details of the trout liver S9 assay results

The five alkylsulfonate homologues tested display a strong chain-length dependency of the clearance rate (Table S17). $C_{10}SO_3$ and $C_{11}SO_3$ showed rapid clearance (689 and 464 mL h^{-1} g liver^{-1}, respectively), with the parent concentration reduced by two orders of magnitude within 30-60 minutes. In comparison to $C_{10}SO_3$, $C_{13}SO_3$ shows a tenfold lower clearance rate for (68 mL h^{-1} g liver^{-1}). The one CH_2 unit longer analogue $C_{14}SO_3$ did not show significant clearance over the course of 60 minutes, and neither did $C_{16}SO_3$ during 120 minute exposure to the active RT-S9 reaction mixture. The decrease of RT-S9 clearance rate with longer chain-length analogues was also described recently for a series of *N,N*-dimethylalkylamines and *N*-methylalkylamines (Droge et al. 2021), but for these structures the C_{16} analogue still showed a significant clearance rate and the difference with C_{10} analogue was only about a factor of 4 lower.

The lower limit of the RT-S9 assay to detect clearance for a given experiment (LL_{S9}) depends on factors specific to the study (e.g., total run time, the number of sampling times, and the number of replicates at each time point) as well as variability in replicated sampling measurements. Using simulation analysis, the lowest detectable depletion rate constant for a 2h RT-S9 assay was determined to be approximately 0.15 h^{-1} for ionizable organic chemicals measured by LC-MS/MS (Chen et al., 2016). For a solution containing 2 mg mL^{-1} S9 protein, this corresponds to a $CL_{int,in\ vivo}$ of approximately 10.5 mL h^{-1} g liver^{-1}. Since the shorter analogues for $C_{14}SO_3$ and $C_{16}SO_3$ did show clearance, it is considered unlikely that the absence of a depletion curve slope significantly different from 0 means that these longer chained analogues are not biotransformed at all in the RT-S9 reaction mixture. For this reason, the $CL_{int,in\ vivo}$ for $C_{14}SO_3$ and $C_{16}SO_3$ was set to LOD/3, i.e. a reaction rate of 0.05 h^{-1}, or ~3.5 mL h^{-1} g liver^{-1} for the extrapolation to a whole body biotransformation rate constant (see Text S4).

A recent study by Nichols et al. (2021) indicates that the addition of the protease-inhibitor phenylmethylsulfonyl fluoride (PMSF) can increase the working lifetime of the trout liver S9 substrate depletion assay, which would result in improved detection of low intrinsic clearance rates. The additional of PMSF is unfortunately not further evaluated for this study, as the research project ended before the work of Nichols et al. was published. For future modelling purposes or regulatory requirements, RT-S9 with addition of PMSF could provide valuable confirmation of the actual *in vivo* intrinsic clearance rates, or absence of a relevant elimination rate via biotransformation, for the longer chained anionic surfactants $C_{14}SO_3$ and $C_{16}SO_3$.

The main objective of the AnionMixture RT-S9 test was to provide a first indication of whether analogue co-solutes can influence the intrinsic clearance of anionic surfactants, but it also provides further supporting confirmation on the measurement of clearance rate for each compound in single assays. Since the *in vivo* BCFs for anionic surfactants were determined using mixtures of anionic surfactants, the RT-S9 clearance rates determined for single chemicals may overestimate enzyme reactivity in the liver of fish exposed to a mixture. The results with the AnionMixture (Table S18) confirmed the absence of significant clearance for BEHP, and clearance close to the lower limit of detection ($CL_{int,\ in\ vivo}$ <10 mL h^{-1} g liver $^{-1}$) for $C_{14}SO_3$ and $C_{16}SO_3$. This provides further support for the selection of a clearance rate of LOD/3 as described above for $C_{14}SO_3$ and $C_{16}SO_3$.

The $CL_{int,in\ vivo}$ for $C_{13}SO_4$ in the AnionMixture was comparable to that tested individually, with the mixture test spanning a full 2 h time series while the individual RT-S9 assay only had quadruplicate samples until 40 minutes. Also, clearance rates in the AnionMixture for DOSS and C_{12}-EO_4-SO_4 were comparable to those in individual RT-S9 assays. Clearance rates were

significantly lower in the AnionMixture for $C_{11}SO_4$ and C_{10}-1-LAS (twofold) and $C_{10}SO_3$ (factor of 6) compared to values obtained in single compound assays (Figure S3 and Table S17).

The presented data thus provide a first indication that anion surfactants may impact the *in vivo* biotransformation of analogue co-solutes with which they often co-exist in technical formulations. On the other hand, initial concentrations differed somewhat compared to single solute tests (Table S17 and Table S18), and further studies on the effect of initial surfactant concentrations would be recommended (Lo et al. 2015; Saunders et al. 2019). It would remain very difficult, though, to assess the most relevant mixture composition in the S9 assay to appropriately resemble the dynamic *in vivo* situation. It remains to be established how the complex compound specific surfactant-enzyme interactions in the *in vitro* assay relate to the *in vivo* observation that the hepatic clearance rate of freely dissolved chemical was similar for the studied surfactants.

References:

Chen, Y.; Hermens, J.L.M.; Jonker, M.T.O.; Armitage, J.M.; Arnot, J.A.; Nichols, J.W.; Fay, K.A.; Droge, S.T.J. Which molecular features affect the intrinsic hepatic clearance rate of ionizable organic chemicals in fish? *Environ. Sci. Technol.* **2016,** *50,* 12722-12731.

Droge, S. T. J.; Armitage, J. M.; Arnot, J. A.; Fitzsimmons, P. N.; Nichols, J. W. Biotransformation potential of cationic surfactants in fish assessed with rainbow trout liver S9 fractions. **2021** (under review)

Lo J. C.; Allard G. N.; Otton S. V.; Campbell D. A.; Gobas F. A. P. C. Concentration dependence of biotransformation in fish liver S9: Optimizing substrate concentrations to estimate hepatic clearance for bioaccumulation assessment. *Environ. Toxicol. Chem.* **2015**, *34,* 2782-2790.

Nichols J. W.; Hoffman A. D.; Swintek J.; Droge S. T. J.; Fitzsimmons P. N. Addition of phenylmethylsulfonyl fluoride increases the working lifetime of the trout liver S9 substrate depletion assay, resulting in improved detection of low intrinsic clearance rates. *Environ. Toxicol. Chem.* **2021**, *40,* 148-161.

Saunders L. J.; Fontanay S.; Nichols J. W.; Gobas F. A. P. C. Concentration dependence of in vitro biotransformation rates of hydrophobic organic sunscreen agents in rainbow trout S9 fractions: Implications for bioaccumulation assessment. *Environ. Toxicol. Chem.* **2019**, *38,* 548-560.

Text S7: Model of biotransformation of ionic surfactants in fish

Our model is based on the venous equilibrium liver model as described in Rowland et al. (1973) and applied to IVIVE in fish by Nichols et al. (2006). We assume that biotransformation occurs primarily in the liver of the fish and that the liver behaves as a well-mixed reactor. We then write the mass balance equation as:

$$Q_B \cdot C_{Bi} = Q_B \cdot C_{Bo} + Q_{LB} \cdot C_{Bo} \quad (1)$$

where Q_B is blood flow through the liver (L h^{-1}), C_{Bi} and C_{Bo} are concentration in blood flowing into and out of the liver, respectively (mol L^{-1}), and Q_{LB} is a clearance rate for the liver expressed in terms of blood flow (L blood h^{-1}). We choose to express the clearance rate in liver in terms of the freely dissolved concentration, as we hypothesize that it is the freely dissolved concentration that determines the interaction with enzymes and that the freely dissolved concentration in venous blood and at the site of metabolism are the same. In this case, Eq. 1 becomes:

$$Q_B \cdot C_{Bi} = Q_B \cdot C_{Bo} + \frac{Q_{LW}}{D_{BW}} \cdot C_{Bo} \quad (2)$$

where Q_{LW} is the clearance rate in liver in terms of the freely dissolved concentration (L h^{-1}) and D_{BW} is the blood-water distribution ratio (L water L^{-1} blood). Assuming that membranes dominate the sorption of anionic surfactants in blood, D_{BW} can be expressed in terms of the membrane lipid-water distribution ratio D_{MLW} (L water kg^{-1} membrane):

$$D_{BW} = f_{WB} + f_{MLB} \cdot D_{MLW} \quad (3)$$

where f_{WB} is the volume fraction of water in blood (L water L^{-1} blood) and f_{MLB} is the mass:volume fraction of membrane lipid in blood (kg L^{-1}). Substituting (3) into (2):

$$Q_B \cdot C_{Bi} = Q_B \cdot C_{Bo} + \frac{Q_{LW}}{f_{WB} + f_{MLB} \cdot D_{MLW}} \cdot C_{Bo} \quad (4)$$

Solving for C_{Bo}:

$$C_{Bo} = \frac{Q_B \cdot C_{Bi}}{Q_B + \frac{Q_{LW}}{f_{WB} + f_{MLB} \cdot D_{MLW}}} \quad (5)$$

To obtain the rate of loss of chemical from the fish due to biotransformation (n_B), we multiply Eq. 5 by the expanded expression for Q_{LB}:

$$n_B = \frac{Q_B \cdot \frac{Q_{LW}}{f_{WB} + f_{MLB} \cdot D_{MLW}} \cdot C_{Bi}}{Q_B + \frac{Q_{LW}}{f_{WB} + f_{MLB} \cdot D_{MLW}}} \quad (6)$$

To convert rate of loss (n_B) to a rate constant for loss, we require the total amount of chemical in the fish N_F (mol):

$$N_F = V_D \cdot C_{Bi} \quad (7)$$

where V_D is the volume of distribution (L). Dividing (6) by (7) gives the whole-body rate constant for loss from the fish due to biotransformation k_B (h^{-1}):

$$k_B = \frac{Q_B \cdot \frac{Q_{LW}}{f_{WB} + f_{MLB} \cdot D_{MLW}}}{Q_B + \frac{Q_{LW}}{f_{WB} + f_{MLB} \cdot D_{MLW}}} \cdot \left(\frac{1}{V_D}\right) \quad (8)$$

f_{WB} is only of interest when Q_B is larger than the second term in the denominator (otherwise the second term in the numerator and denominator cancel each other). For this to be true, (f_{WB} + $f_{MLB}{\cdot}D_{MLW}$) must be large, which means f_{WB} is negligible. Eq. 8 can thus be simplified to:

$$k_B = \frac{Q_B \cdot \frac{Q_{LW}}{f_{MLB} \cdot D_{MLW}}}{Q_B + \frac{Q_{LW}}{f_{MLB} \cdot D_{MLW}}} \cdot \left(\frac{1}{V_D}\right) \quad (9)$$

Two cases can be defined:

I) Blood flow is rate limiting, $Q_B \ll \frac{Q_{LW}}{f_{MLB} \cdot D_{MLW}} \;\rightarrow\; k_B = \left(\frac{Q_B}{V_D}\right)$ (10)

II) Transformation in liver is rate limiting, $Q_B \gg \frac{Q_{LW}}{f_{MLB} \cdot D_{MLW}} \;\rightarrow\; k_B = \frac{Q_{LW}}{f_{MLB} \cdot D_{MLW}} \cdot \left(\frac{1}{V_D}\right)$ (11)

In Case I we obtain the intuitive result that k_B is equal to the quotient of the hepatic blood flow rate and the volume of distribution. Note that k_B is a constant, independent of D_{MLW}. This case occurs where D_{MLW} is small.

In Case II k_B is inversely proportional to D_{MLW}; as D_{MLW} increases k_B decreases below the maximum value defined by Case I. If Q_{LW} is constant then D_{MLW} will be the only variable that influences k_B in a given fish (i.e., with fixed f_{MLB} and V_D).

Note also that inverting Eq. 9 gives the simpler relationship:

$$\frac{1}{k_B} = V_D \cdot \left(\frac{f_{MLB} \cdot D_{MLW}}{Q_{LW}} + \frac{1}{Q_B}\right) \quad (12)$$

<u>References</u>

Nichols, J. W.; Schultz, I. R.; Fitzsimmons, P. N. In vitro–in vivo extrapolation of quantitative hepatic biotransformation data for fish I. A review of methods, and strategies for incorporating intrinsic clearance estimates into chemical kinetic models. *Aquat. Toxicol.* **2006**, *78*, 74-90.

Rowland, M.; Benet, L. Z.; Graham, G. G. Clearance concepts in pharmacokinetics. *J Pharmacokinet. Biopharm.***1973**, *1*, 123-136.

Table S1: Anionic surfactants with tonnage >100/y in the EU (REACH data aq. surface tension <65 mN/m). Red print in the column *Anion type* indicates that the substance is similar to one of the test chemicals in this study.

Tonnage (y^{-1})	CAS	Anion type	Chain length range ("average")	Substance name (with hyperlink to REACH dossier)	Surface tension	REACH dossier #	Degradation information ~28d-sludge test Orange: lower degradability	Examples of BCF info in REACH-dossier [a] Green: *in vivo* BCF study included
100.000-1.000.000	8002-26-4	CO2	C14 - sterol	Tall oil	63.5	14967	60-64% degradable	
100.000-1.000.000	65997-01-5	CO2	C14 - sterol	Tall oil, sodium salt	55.8	15402	78-83% degradable	
100.000-1.000.000	8016-81-7	CO2	C14 - sterol	Tall-oil pitch	64.9	15121	9.2-36% degradable	
10.000-100.000	85586-07-8	SO4	C12-14 (13)	Sulfuric acid, mono-C12-14-alkyl esters, sodium salts	29.9	16003	98% degradable	LogKow ≤ -2.42
10.000-100.000	68955-19-1	SO4	C12-18 (15)	Sulfuric acid, mono-C12-18-alkyl esters, sodium salts	31.9	15009	73->94% degradable	LogKow ≤ -2.1
10.000-100.000	161074-79-9	SLES	C12-13 (12)	Alcohols, C12-13, branched and linear, ethoxylated, sulfates, sodium salts	28.24	14953	65-100% degradable	
10.000-100.000	97489-15-1	SO3	C14-17 (15.2)	Sulfonic acids, C14-17-sec-alkane, sodium salts	34	13425	69-95% degradable	logKow = 0.2, and/or a low potential to cross biological membranes
10.000-100.000	68188-18-1	SO3	C7-14 (10)	Paraffin oils, sulfochlorinated, saponified	35.4	2228	82-99% degradable	
10000-100.000	68937-75-7	CO2	C8-10 (9)	Fatty acids, C8-10	33.7	15345	62-86% degradable	Reference to *in vivo* BCF: 255 L/kg for (for C11CO2) Danio rerio 28d pH7.6)
10.000-100.000	65997-03-7	CO2	C16-18 (17)	Fatty acids, tall-oil, low-boiling	63.2	14791	41% degradable	Data waiving: study scientifically not necessary
10.000+	577-11-7	SO3-DOSS	C8x2	sodium 1,4-bis[(2-ethylhexyl)oxy]-1,4-dioxobutane-2-sulfonate	30.65	16066	91.2-100% degradable	LogKow is 2. Since furthermore the substance shows ready biodegradation, persistency in the environment is unlikely. In conclusion, bioaccumulation of Docusate Sodium is of no concern.
100-1000	7491-09-0	SO3 - DOSS	C8 x 2	potassium 1,4-bis[(2-ethylhexyl)oxy]-1,4-dioxobutane-2-sulfonate	30.65	10211	83.5-100% degradable	LogKow = 2.0 (solubility ratio)
10.000+	3302-10-1	CO2 branched	C9	3,5,5-trimethylhexanoic acid	35.3	13645	96% degradable	Reference to *in vivo* BCF: BCF 1-7 L/kg (carp 28d pH7)
10.000+	7620-77-1	CO2 hydroxy	C18	lithium(1+) ion 12-hydroxyoctadecanoate	34/52	15435	74-97% degradable	LogKow < 3
1000-10.000	12645-31-7	PO4 - BEHP	C8	[(2-ethylhexyl)oxy]phosphonic acid	36.03	13106	26-78% degradable	LogKow < 3
1000-10.000	151-21-3	SO4	C12	sodium dodecyl sulfate	25.2	2126	94-97% degradable	Experimental several studies BCF~1-10
1000-10.000	73296-89-6	SO4	C12-16 (14)	Sulfuric acid, mono-C12-16-alkyl esters, sodium salts	31.9	13301	100% degradable	LogKow ≤ -2.1
1000+	68610-66-2	SLES	C10-12	Sulfated Alcohols, C10-12 (even numbered), ethoxylated (1-2.5 EO), sulfated, sodium salts	34	5817	78-100% degradable	Ref to AEO nonionic
1000-10.000	61789-32-0	SO3 acetate	C12-14 (13)	Fatty acids, coco, 2-sulfoethyl esters, sodium salts	24	14398	78% degradable	LogKow = -0.41
1000-10.000	EC 932-051-8	SO3 Benzyl	C10-13 (12)	Reaction product of Benzenesulfonic acid, 4-C10-13-sec-alkyl derivs. and Benzenesulfonic acid, 4-methyl- and sodium hydroxide	ca. 34.8	10765	49-94% degradable	Exp BCF 2 - 987 L/kg (Tolls)
1000-10.000	EC 939-185-6	SO3 Benzyl	C20-24 (22)	Benzenesulfonic acid, Methyl-, Mono-C20-24-(even numbered)-branched Alkyl derivs	35.8	18545	8% degradable	LogKow = 10.60 (?), in BCFBAF

1000-10.000	91696-74-1	SO3	C14-44 (29) branched	Benzenesulfonic acid, C14-44-branched	68	2231	1.5-9.1% degradable	Exp BCF 104 L/kg (bluegill 14C-LAS)
1000-10.000	64754-95-6	CO2 hydroxy	C18+	dilithium(1+) ion 12-hydroxyoctadecanoate octadecanoate	34/52	14556	74-97% degradable	LogKow < 3
1000-10.000	112-05-0	CO2	C9	nonanoic acid	31.7	13098	68 -75% degradable	BCFBAF: 3.2 L/kg (logKow of 3.42)
1000-10.000	90990-10-6	CO2	C12-14 (13)	Fatty acids, C12-14	32	16064	>55-85% degradable	Exp BCF 255 for (for C11CO2) Danio rerio 28d pH7.6)
1000-10.000	71076-48-7	CO2	C8-24	Fatty acids, C8-24	32	21079	no data	Exp BCF 255 for (for C11CO2) Danio rerio 28d pH7.6)
1000-10.000	18777-32-7	CO2 amido	C8-14 (11)	Fatty acid chlorides, C8-14 (even numbered), reaction products with glycine	23.2	13235	83—86% degradable	Log Kow = -0.2
1000-10.000	91722-33-7	CO2	sterol - C14	Tar, wood	60.24	5650	47% degradable	LogKow < 2.02
100-1000	4724-48-5	PO3	C8	octylphosphonic acid	30.4	14433	65-88% degradable	LogKow = 2.7(pH1)
100-1000	154518-38-4	PO4	C11-14 dialkyl	Phosphoric acid, C11-14-isoalkyl esters, C13-rich	38.1	12215	22% degradable	LogKow = 2.18 (± 0.16) (pH estimated to be ≥4)
100-1000	90506-73-3	PO4	C16-18	Phosphoric acid, mono- and di-C16-18 (even numbered) alkyl esters	60.6	5908	15% degradable	LogKow = 2.5
100-1000	39322-78-6	PO4	C12	potassium dodecyl hydrogen phosphate	≥43.9	5556	4-63% degradable	LogKow = 2.74 (solubility ratio)
100-1000	68987-29-1	PO4	C18	1-Octadecanol, phosphate, potassium salt	≥ 67.6	5523	0% degradable	LogKow of Silastol H 200-TS is calculated as 8.556 (HPLC)
100-1000	13360-78-6	PO4	C4x2 isobutyl	sodium diisobutylphosphinodithioate	68.7	12303	78,8% degradable	LogKow = -1.64
100-1000	84501-49-5	SO4	C9-11 (10)	Sulfuric acid, mono-C9-11-alkyl esters, sodium salts	23.3	17155	95% degradable	LogKow < 3 (log Pow = 1.057 at 23.5 °C, pH 6.92-7.01 by shake flask method)
100-1000	90583-16-7	SO4	C12-14 (13)	Sulfuric acid, mono-C12-14 (even numbered)-alkyl esters	22.3	12069	71-87% degradable	LogKow < 3
100-1000	156572-81-5	SO4	C12 amido	disodium (2R)-2-(dodecanoyloxy)propane-1-sulfonate (2S)-2-(dodecanoyloxy)propane-1-sulfonate	ca. 38.6	22826	83.9% degradable	Exposure is unlikely
100-1000	85681-55-6	SO3 acetate	C12-16 (14)	Acetic acid, 2-sulfo, mono-C12-14(even numbered)-alkylesters, sodium salt	32	5420	70% degradable	LogKow = -0.3
100-1000	1258274-08-6	SO3	C9 x 2 + naphthyl	Reaction products of aromatic hydrocarbons, C10-13 with branched nonene, sulfonated, sodium salts	30	12148	49% degradable	LogKow = -3.3
100-1000	68609-93-8	SO3	C18	9-Octadecenoic acid (Z)-, sulfonated, potassium salts	32.5	10668	66% degradable	Via log D in BCFBAF
100-1000	84753-04-8	CO2	C14-18 (16)	fatty acids C14-18/C16-18 unsaturated with dihydrogendioxide and Ammonia	42.9	10627	83-93% degradable	LogKow = 1.48 (shake flask)
100-1000	71902-23-3	CO2 amido	C8 + C6	6-[(1-formyloctyl)amino]hexanoic acid	≥ 40.1	11335	98% degradable	LogKow = 2.47 (shake flask)
100-1000	1424149-03-0	CO2 amido ethanol	C13-16 (15)	4-((2-hydroxyethyl)amino)-3-pentaproenyl-4-oxobutanoate - sodium/triethanolamine	≥ 37.9	6386	53% degradable	LogKow = 1.1 at pH 7
100-1000	EC 946-061-5	CO2 - EO	C16-18 (17)	Poly(oxy-1,2-ethanediyl), α-(carboxymethyl)-ω-hydroxy-, C16-18 (even numbered) and C18-unsatd. alkyl ethers	29.8	20396	73-90% degradable	LogKow = 4.77
100+	68783-37-9	CO2	C16-18 (17)	Fatty acids, C16-18, lithium salts	34	5899	75-95% degradable	LogKow values of <3

[a] To go to the dossier, click the hyperlink in the 5th column, or add the dossier-number in column 7 in the following link at X [https://echa.europa.eu/nl/registration-dossier/-/registered-dossier/X] to retrieve the information submitted on Bioaccumulation in the section on Environmental fate & pathways.

Table S2: Test chemical standards

Abbr.	Name	CAS #	Supplier	Purity (%)
$C_{10}SO_3$	Decylsulfonate	13419-61-9	Fluka	>99
$C_{11}SO_3$	Undecylsulfonate	5838-34-6	Research Plus	N.A.
$C_{13}SO_3$	Tridecylsulfonate	5802-89-1	Research Plus	N.A.
$C_{14}SO_3$	Tetradecylsulfonate	6994-45-2	TCI	>98
$C_{16}SO_3$	Hexadecylsulfonate	15015-81-3	TCI	N.A.
$C_{11}SO_4$	Undecylsulfate	1072-24-8	Research Plus	N.A.
$C_{13}SO_4$	Tridecylsulfate	3026-63-9	Research Plus	N.A.
C_{10}-1-LAS	1-n-(p-sulfophenyl)decane	140-60-3	Synth. by J. Tolls[#]	N.A.
C_{12}-EO_4-SO_4	Dodecyltetraethoxysulfate	9004-82-4	Supplied by P&G	>95
DOSS	Bis(2-ethylhexyl)-sulfosuccinate	577-11-7	Sigma	>99
BEHP	Bis(2-ethylhexyl)phosphate	298-07-7	Aldrich	97
$C_{12}SO_4$-IS	D_{25}-dodecylsulfate	110863-24-6	TRC	98
BEHP-IS	D_{34}-bis(2-ethylhexyl)-phosphate	1773493-20-1	TRC	97

[#] Tolls, J. Bioconcentration of Surfactants. PhD, Utrecht University, Utrecht, 1998

Table S3: Solvents

Solvent	Purpose	Grade	Supplier
Methanol	Test chemical solution for aquaria, extraction, UPLC mobile phase	Lichrosolve	Merck
Milli-Q water	Dilution of purified extract, UPLC mobile phase		
Ammonium acetate	UPLC mobile phase	p.a.	Merck

Table S4: Sampling schedule

Day	hours	Water	Fish	Alkalinity	TOC
1	0	X	X	X	X
1	0.5	X			
1	1.2	X	X		X
1	2.1	X	X		
1	3.1	X	X		
1	4.1	X	X		
1	6.1	X	X		
2	24.1	X	X	X	X
2	29.2	X			
3	51.4	X	X	X	X
3	55.0	X			
4	74.0	X	X	X	X
4	78.9	X			
5	96.0	X	X	X	X
Transfer fish to elimination aquarium					
5	96.9	X	X		
5	97.9	X	X		
5	100.0		X		
5	104.2	X	X		
5	106.0		X		X
6	119.3	X	X	X	
6	128.0		X		
7	144.8	X	X	X	
8	168		X	X	X
9	192.0		X		
12	264.0		X		

Table S5: Liquid chromatography program

Time (min)	Flow (mL min^{-1})	%A	%B
0	0.3	90	10
2	0.3	50	50
4	0.3	35	65
6	0.3	35	65
10	0.3	10	90
10.2	0.5	2	98
11.5	0.5	2	98
12.5	0.5	90	10

Table S6: MS/MS MRM parameters. CE is collision energy (eV). Source parameters were set to: Source Type, H-ESI; spray negative, 4000 V; sheath gas, 45; aux gas, 20; sweep gas, 0.5; ion transfer tube temp, 350 °C; vaporizer temp, 350 °C; Q1 & Q3 resolution, 0.7; source fragmentation, 0 V; collision gas pressure, 1.5 mTorr.

Compound	CE	Parent ion (m/z)	Daughter ion (m/z)	QuantIon
BEHP (IS) D_{34}	33.76	355.6	79	QuantIon
BEHP (IS) D_{34}	41.35	355.6	207.097	
BEHP (IS) D_{34}	22.99	355.6	227.169	
SDS (IS) D_{25}	55	290.5	80	
SDS (IS) D_{25}	55	290.5	96.089	
SDS (IS) D_{25}	31.99	290.5	97.929	QuantIon
$C_{10}SO_3$	28.46	221.3	80.071	QuantIon
$C_{10}SO_3$	10.25	221.3	135.222	
$C_{10}SO_3$	22.74	221.3	155.222	
$C_{11}SO_3$	29.52	235.3	80	QuantIon
$C_{11}SO_3$	23.7	235.3	169.222	
$C_{11}SO_3$	22.34	235.3	191.238	
$C_{13}SO_3$	30.33	263.3	79.986	QuantIon
$C_{13}SO_3$	39.63	263.3	107.014	
$C_{13}SO_3$	23.4	263.3	197.165	
$C_{14}SO_3$	32.5	277.3	80	QuantIon
$C_{14}SO_3$	28.3	277.3	81.026	
$C_{14}SO_3$	50.4	277.3	93.982	
$C_{16}SO_3$	33.56	305	80	QuantIon
$C_{16}SO_3$	46.2	305	94.058	
$C_{16}SO_3$	24.97	305	293.294	
$C_{11}SO_4$	51.87	251.2	80.071	
$C_{11}SO_4$	27.7	251.2	96.986	QuantIon
$C_{11}SO_4$	32.9	251.2	164.986	
$C_{12}EO_4SO_4$	46.1	441.3	80	
$C_{12}EO_4SO_4$	36.95	441.3	97	QuantIon
$C_{12}EO_4SO_4$	42.71	441.3	211.071	
$C_{13}SO_4$	48.88	279.2	80.058	
$C_{13}SO_4$	29.01	279.2	97	QuantIon
$C_{13}SO_4$	17.99	279.2	261.071	
C_{10}-1-LAS	33.61	297	170	
C_{10}-1-LAS	37.05	297	183.071	
C_{10}-1-LAS	26.79	297	233.151	
BEHP	30.07	321	78.986	
BEHP	40.08	321	165.058	
BEHP	19.71	321	209.04	
DOSS	26.69	421.3	80.968	QuantIon
DOSS	20.11	421.3	227.022	
DOSS	21.17	421.3	291.058	

Table S7: Characterization of the liver S9 batch, and comparison to another batch pooled from the same liver homogenate

	**Batch used for anionic surfactants** [a]	**Batch used for cationic surfactants (Droge et al. 2021b)**
**Extrapolation scaling factors for S9 fractions**		
CYP content in S9 fraction (pmol CYP g liver $^{-1}$)	4728	4242
CYP content in liver homogenate (pmol CYP g liver $^{-1}$)	13604	13098
Recovery of CYP (%)	34.8	32.4 [b]
Protein content of RT-S9 (mg protein mL RT-S9 $^{-1}$)	23.8	23.1
S9 content of liver (ml S9 g liver $^{-1}$)	2.16	2.13
S9 content of liver (mg S9 protein g liver $^{-1}$)	147.8	152.0
**Activity of S9 fractions**		
CYP content (pmol P450 mg protein^{-1})	91.8 ± 6.3	86.4 ± 6.5
EROD activity (pmol min^{-1} · mg protein)	3.9 ± 0.2	3.7 ± 0.4
UGT activity (pmol min^{-1} · mg protein)	1179 ± 6	1168 ± 74
GST activity (pmol min^{-1} · mg protein)	501 ± 18	508 ± 46

[a] Variances are reported as ± standard deviation.

[b] Calculated as described by Nichols et al. (2013b).

CYP = cytochrome P450; EROD = 7-ethoxyresorufin O-deethylase; UGT = UDPGA-glucuronosyltransferase; GST = glutatione S-transferase.

Table S8: Mean relative standard deviation of test substance concentration in triplicate water samples collected during the exposure phase (n = 14 × 3)

**CHEMICAL**	**Mean RSD (%)**
$C_{10}SO_3$	3
$C_{11}SO_3$	3
$C_{13}SO_3$	6
$C_{14}SO_3$	12
$C_{16}SO_3$	24
$C_{11}SO_4$	4
$C_{13}SO_4$	10
C_{10}-1-LAS	9
C_{12}-EO_4-SO_4	16
DOSS	25

Table S9: Mean relative standard deviation of test substance concentration in a) triplicate injection of the same extract; b) analysis of triplicate extracts of the same fish.

CHEMICAL	Mean RSD Triplicate injection	Mean RSD Triplicate extraction
$C_{10}SO_3$	7	23
$C_{11}SO_3$	4	14
$C_{13}SO_3$	7	8
$C_{14}SO_3$	3	7
$C_{16}SO_3$	not analysed	not analysed
$C_{11}SO_4$	7	14
$C_{13}SO_4$	4	9
C_{10}-1-LAS	4	9
C_{12}-EO_4-SO_4	15	13
DOSS	11	0.4

Table S10: Limit of quantification (LOQ, ng g^{-1}) for fish analysis (determined as mean + 10 × standard deviation of the concentration in control fish)

CHEMICAL	Mean concentration in control fish (ng g^{-1})	LOQ (ng g^{-1})
$C_{10}SO_3$	0.39	2.5
$C_{11}SO_3$	0.48	4.0
$C_{13}SO_3$	0.19	0.88
$C_{14}SO_3$	0.05	0.24
$C_{16}SO_3$	0.43	1.45
$C_{11}SO_4$	0.17	1.6
$C_{13}SO_4$	1.18	5.4
C_{10}-1-LAS	0.12	0.51
C_{12}-EO_4-SO_4	2.1	8.1
DOSS	0.70	3.3

Table S11: Concentrations (µg L^{-1}) of test chemicals in water during the experiment. Each data point is the mean of triplicate samples.

Day	hours	$C_{10}SO_3$	$C_{11}SO_3$	$C_{13}SO_3$	$C_{14}SO_3$	$C_{16}SO_3$
Exposure						
1	0	71	49	6.5	6.1	4.2
1	0.5	68	46	7.9	8.8	6.3
1	1.2	59	41	6.1	7.3	6.6
1	2.1	59	41	6.1	7.0	5.1
1	3.1	61	42	6.6	8.2	5.2
2	4.1	61	43	6.3	8.0	7.3
2	6.1	59	41	6.6	8.0	5.8
2	24.1	56	38	5.2	5.4	5.0
3	29.2	61	42	6.5	8.7	11.0
4	51.4	63	44	5.4	6.4	5.5
5	55.0	59	39	5.3	6.6	7.0
6	74.0	50	34	4.2	4.8	5.3
7	78.9	52	36	4.4	5.4	6.0
8	95.1	51	35	3.7	4.1	4.2
Clearance						
9	96.9	0.029	0.017	0.003	0.024	0.149
10	97.9	0.027	0.011	0.008	0.024	0.123
11	104.2	0.009	0.007	0.004	0.011	0.035
12	119.3	0.018	0.022	0.004	0.018	0.005
13	144.8	0.025	0.024	0.004	0.016	0.003
Control aquarium						
14	24	0.025	0.049	0.007	0.013	0.003
15	102	0.027	0.033	0.004	0.015	0.004

Table S11 (continued): Concentrations (µg L^{-1}) of test chemicals in water during the experiment. Each data point is the mean of triplicate samples.

Day	hours	$C_{11}SO_4$	$C_{13}SO_4$	C_{10}-1-LAS	C_{12}-EO_4-SO_4	DOSS
Exposure						
1	0	28	10.9	7.6	32	33
1	0.5	26	15.4	9.9	52	58
1	1.2	21	10.4	7.5	39	52
1	2.1	22	10.1	7.4	41	46
1	3.1	23	12.2	8.2	50	57
2	4.1	24	12.2	8.3	44	70
2	6.1	22	12.2	8.2	52	60
2	24.1	20	8.2	6.2	30	40
3	29.2	22	11.5	8.4	47	87
4	51.4	20	8.0	7.2	41	60
5	55.0	19.0	8.1	7.1	47	66
6	74.0	12.6	4.5	5.5	34	54
7	78.9	12.5	4.4	5.8	37	74
8	95.1	9.9	3.0	5.1	29	45
Clearance						
9	96.9	0.011	0.135	0.018	0.291	0.146
10	97.9	0.008	0.014	0.010	0.018	0.082
11	104.2	0.025	0.006	0.003	0.035	0.108
12	119.3	0.049	0.003	0.003	0.011	0.010
13	144.8	0.006	0.009	0.000	0.009	0.015
Control aquarium						
14	24	0.007	0.020	0.001	0.037	0.031
15	102	0.002	0.007	0.000	0.013	0.009

Table S12: Variability of test substance concentration in fish samples at a given time point, expressed as the RSD for the 3 fish at each time point, averaged for all time points where the concentration in all 3 fish was above the LOQ: a) including all data; b) after removal of outliers.

CHEMICAL	Mean RSD (%) (all data)	Mean RSD (%) (outliers removed)
$C_{10}SO_3$	NQ#	NQ#
$C_{11}SO_3$	21	21
$C_{13}SO_3$	38	26
$C_{14}SO_3$	32	25
$C_{16}SO_3$	23	20
$C_{11}SO_4$	31	20
$C_{13}SO_4$	23	21
C_{10}-1-LAS	20	18
C_{12}-EO_4-SO_4	19	19
DOSS	33	29

there were too few data above the LOQ to allow quantification of the mean RSD.

Table S13a: Concentrations (ng g^{-1} ww) of test chemicals in fish during the exposure phase.

Day	hours	$C_{10}SO_3$	$C_{11}SO_3$	$C_{13}SO_3$	$C_{14}SO_3$	$C_{16}SO_3$
Exposure						
1	0	0.2	0.50	0.16	0.06	0.30
1	0	0.3	0.16	0.04	0.03	0.27
1	0	0.2	0.15	0.10	0.02	0.55
1	1	1.1	3.2	15.3	38	440
1	1	2.1	1.5	2.5	7.5	319
1	1	1.4	8.4	35	72	614
1	2	1.1	2.1	3.2	11.3	300
1	2	7.5	1.9	3.0	11.7	304
1	2	1.1	1.4	2.3	6.8	147
1	3	1.5	2.9	4.7	16.0	347
1	3	1.6	3.6	5.8	19.9	392
1	3	5.5	3.2	4.5	15.9	451
1	4	2.4	3.5	8.1	27	510
1	4	3.5	13.4	54	153	1310
1	4	6.5	41	202	415	2283
1	6	2.1	4.7	10.5	41	557
1	6	2.0	5.0	10.3	39	678
1	6	1.4	3.8	8.5	32	649
2	24	42.8	5.5	24	101	2045
2	24	1.4	4.2	21	111	1698
2	24	1.4	3.8	17.0	80	1479
3	48	2.4	4.4	19.4	152	3941
3	48	3.6	5.1	20	155	3596
3	48	1.7	3.7	17.1	117	1879
4	72	3.6	7.2	30	180	1532
4	72	2.1	5.2	16.7	141	3782
4	72	3.0	4.9	15.0	103	3167
5	96	3.0	8.1	22	168	3775
5	96	3.0	7.8	19.7	150	5248
5	96	2.8	5.3	28	178	3695

< LOQ

Other fish at same time point < LOQ

Outlier

Table S13a (continued): Concentrations (ng g^{-1} ww) of test chemicals in fish during the exposure phase.

Day	hours	$C_{11}SO_4$	$C_{13}SO_4$	C_{10}-1-LAS	C_{12}-EO_4-SO_4	DOSS
Exposure						
1	0	0.02	0.4	0.02	1.2	0.50
1	0	0.11	1.6	0.04	2.0	0.58
1	0	0.07	1.8	0.03	2.0	1.00
1	1	3.1	34	8.9	4.3	10.9
1	1	3.6	28	6.9	6.7	5.8
1	1	4.1	54	16.6	6.6	17.8
1	2	5.6	40	11.3	6.7	8.5
1	2	4.4	42	10.6	8.9	11.0
1	2	4.1	27	7.2	6.4	7.5
1	3	7.5	61	16.5	12.1	14.9
1	3	9.4	67	19.7	10.4	17.4
1	3	7.6	60	16.6	10.6	15.2
1	4	10.7	88	26	14.5	21
1	4	11.2	108	32	16.2	42
1	4	18.0	187	75	14.2	106
1	6	15.8	139	38	23	36
1	6	14.7	132	38	18.6	32
1	6	14.1	97	27	13.5	25
2	24	18.5	255	86	24	66
2	24	15.1	255	91	34	59
2	24	10.9	188	64	23	41
3	48	7.4	140	94	26	48
3	48	12.0	234	98	33	66
3	48	11.2	190	84	38	56
4	72	12.6	201	112	30	50
4	72	8.7	154	87	39	59
4	72	8.2	128	70	35	78
5	96	9.9	175	113	45	84
5	96	8.7	150	104	27	62
5	96	14.1	275	110	42	67

< LOQ

Other fish at same time point < LOQ

Outlier

Table S13b: Concentrations (ng g^{-1} ww) of test chemicals in fish during the elimination phase.

Day	hours	$C_{10}SO_3$	$C_{11}SO_3$	$C_{13}SO_3$	$C_{14}SO_3$	$C_{16}SO_3$
Elimination						
5	97	3.0	5.5	15.7	113	4206
5	97	1.5	3.8	14.6	118	4275
5	97	1.3	3.2	19.2	164	4280
5	98	1.5	3.2	14.6	130	3644
5	98	1.0	2.8	12.4	119	3202
5	98	1.4	30.6	14.8	109	3035
5	100	5.1	14.1	33	185	4355
5	100	1.4	2.8	10.3	93	3469
5	100	1.4	3.1	12.1	114	3603
5	103	0.3	2.2	10.0	94	3251
5	103	0.3	3.0	23	194	4192
5	103	0.6	6.7	11.1	108	3692
5	106	286	231	71	159	3481
5	106	2.0	2.7	11.9	113	2894
5	106	2.5	9.3	57	240	4335
6	120	0.50	0.58	6.4	62	2630
6	120	0.76	0.87	4.3	81	4641
6	120	0.61	0.78	2.9	50	2805
6	128	0.62	0.53	2.8	60	3635
6	128	0.49	0.50	2.3	45	2712
6	128	0.99	0.58	4.9	68	2831
7	144	0.63	0.49	1.63	42	2859
7	144	0.33	0.42	1.18	31	2503
7	144	5.2	0.53	1.59	70	3401
8	168	0.49	0.56	0.83	30	2804
8	168	0.55	0.41	1.89	28	2276
8	168	0.16	0.22	0.64	16.5	2244
9	192	0.14	0.10	0.40	11.9	1669
9	192	0.15	0.19	0.33	8.6	1852
9	192	0.22	0.19	0.60	12.7	2440
12	264	2.0	1.49	0.30	6.6	1420
12	264	1.9	0.23	0.13	4.8	2150
12	264	0.2	0.19	0.02	1.75	967

< LOQ

Other fish at same time point < LOQ

Outlier

Table S13b (continued): Concentrations (ng g^{-1} ww) of test chemicals in fish during the elimination phase.

Day	hours	$C_{11}SO_4$	$C_{13}SO_4$	C_{10}-1-LAS	C_{12}-EO_4-SO_4	DOSS
Elimination						
5	97	5.9	99	78	35	57
5	97	5.5	120	78	26	69
5	97	6.7	148	103	27	70
5	98	4.6	111	73	17.6	47
5	98	4.4	104	72	19.9	25
5	98	3.8	80	55	15.8	34
5	100	6.6	104	74	35.9	92
5	100	3.0	78	57	14.4	49
5	100	3.5	80	60	21	45
5	103	1.5	58	47	11.6	35
5	103	2.0	80	58	16.1	35
5	103	2.7	82	51	15.1	33
5	106	76	119	85	16.4	93
5	106	3.1	86	65	17.1	37
5	106	2.4	90	59	14.3	53
6	120	1.06	32	24	7.0	14.2
6	120	0.77	40	27	8.6	36
6	120	0.51	25	17.2	3.1	13.5
6	128	0.66	21	14.0	5.5	13.9
6	128	0.48	29	17.0	7.5	15.7
6	128	0.54	21	18.2	4.5	17.8
7	144	0.53	14.4	7.6	4.7	4.1
7	144	0.27	11.1	6.8	5.3	6.4
7	144	0.19	13.5	10.2	4.5	24
8	168	0.59	12.9	4.7	4.1	2.3
8	168	0.22	6.4	3.5	2.7	7.9
8	168	0.17	7.3	3.5	1.8	3.0
9	192	0.08	4.0	1.08	1.6	0.75
9	192	0.13	4.7	1.14	3.6	4.2
9	192	0.09	3.9	1.40	1.8	0.92
12	264	0.84	3.8	1.0	3.3	4.5
12	264	0.15	2.1	0.7	2.4	2.9
12	264	0.15	1.7	0.31	1.5	0.40

< LOQ

Other fish at same time point < LOQ

Outlier

Table S14: Uptake rate constant (k_U), elimination rate constant (k_T) and bioconcentration factor (BCF) of the test chemicals determined using nominal concentrations in water

CHEMICAL	k_U (L kg^{-1} h^{-1})	k_T (h^{-1})	BCF (L kg^{-1} ww)
$C_{10}SO_3$	not quantifiable	not quantifiable	0.046
$C_{11}SO_3$	not quantifiable	not quantifiable	0.137
$C_{13}SO_3$	0.25	0.065	3.9
$C_{14}SO_3$	0.90	0.028	32
$C_{11}SO_4$	0.133	0.233	0.57
$C_{13}SO_4$	2.29	0.074	31
C_{10}-1-LAS	0.74	0.049	15.0
C_{12}-EO_4-SO_4	0.162	0.114	1.42
DOSS	0.169	0.056	3.0

Table S15: Uptake rate constant (k_U), elimination rate constant (k_T) and bioconcentration factor (BCF) of the test chemicals determined from measured concentrations in water using the simpler estimation method described in Text S3. The 95% confidence intervals are given in brackets.

CHEMICAL	k_U (L kg^{-1} ww h^{-1})	k_T (h^{-1})	BCF (L kg^{-1} ww)
$C_{13}SO_3$	0.24 (10)	0.057 (7)	4.3 (12)
$C_{14}SO_3$	0.73 (6)	0.026 (5)	28 (8)
$C_{16}SO_3$	10.8 (10)	0.0073 (10)	1480 (14)
$C_{11}SO_4$	0.133 (27)	0.179 (19)	0.74 (33)
$C_{13}SO_4$	1.64 (15)	0.053 (7)	31 (16)
C_{10}-1-LAS	0.67 (8)	0.046 (3)	14.7 (8)
C_{12}-EO_4-SO_4	0.090 (12)	0.098 (26)	0.92 (29)
DOSS	0.088 (9)	0.064 (11)	1.37 (14)

Table S16: Comparison of the BCF (determined with the measured concentrations in water) and the baseline screening BCF (determined as 0.0125 D_{MLW}[a])

CHEMICAL	Log BCF ($L\ kg^{-1}$ ww)	Log baseline BCF ($L\ kg^{-1}$ ww)	Log difference ($L\ kg^{-1}$ ww)
$C_{10}SO_3$	-1.38	1.11	2.48
$C_{11}SO_3$	-0.87	1.49	2.35
$C_{13}SO_3$	0.65	2.56	1.90
$C_{14}SO_3$	1.47	3.05	1.58
$C_{16}SO_3$	3.14	4.29	1.15
$C_{11}SO_4$	-0.11	2.26	2.37
$C_{13}SO_4$	1.53	3.31	1.78
C_{10}-1-LAS	1.21	3.20	1.99
C_{12}-EO_4-SO_4	-0.05	2.34	2.38
DOSS	0.15	2.68	2.53

[a] Droge, S. T. J.; Scherpenisse, P.; Arnot, J. A.; Armitage, J. M.; McLachlan, M. S.; Nichols, J.; von der Ohe, P.; Bonnell, M.; Hodges, G. Screening the baseline fish bioconcentration potential of various types of surfactants using phospholipid binding data. Environ. Sci.: Processes Impacts 2021, 23, 1930-1948.

Table S17: Starting substrate concentrations, depletion curve fitting parameters and calculated intrinsic clearance rates ($CL_{int,in\ vitro}$ and $CL_{int,in\ vivo}$) for chemicals tested individually or as part of a mixture

CHEMICAL	initial conc. (µM)	Slope	R^2	n	$CL_{int,S9}$ [a] (mL h^{-1} mg $protein^{-1}$)	$CL_{int,in\ vivo}$ (SE) [b] (mL h^{-1} g $liver^{-1}$)
$C_{10}SO_3$	0.1	-0.0657	0.97	12	4.54	670 (36)
$C_{11}SO_3$	1.2	-0.0442	0.97	18	3.05	451 (19)
$C_{13}SO_3$	0.8	-0.0065	0.93	16	0.45	66 (5)
$C_{14}SO_3$	0.4	-0.0016	n.s.	21	n.s.	n.s.
$C_{16}SO_3$	0.1	0.0002	n.s.	18	n.s.	n.s.
$C_{11}SO_4$	1.6	-0.0407	0.98	8	2.81	415 (23)
$C_{13}SO_4$	1.0	-0.0058	0.92	12	0.40	59 (6)
C_{10}-1-LAS	0.3	-0.0089	0.97	12	0.61	91 (5)
C_{12}-EO_4-SO_4	0.3	-0.0188	0.77	15	1.30	192 (30)
DOSS	2.2	-0.0021	0.89	12	0.14	21 (2)
BEHP	3.1	-0.0001	n.s.	12	n.s.	n.s.

[a] The measured recovery scaling factor of 147.8 mg RT-S9 protein g $liver^{-1}$ was used.

[b] $CL_{int,in\ vivo}$ values are reported as the calculated value with standard error between brackets (SE). The SE of $CL_{int,in\ vivo}$ was calculated from the SE of the fitted regression slope.

n.s. = depletion slope not significantly different from 0.

Table S18: Starting substrate concentrations, depletion curve fitting parameters and calculated intrinsic clearance rates ($CL_{int,in\ vivo}$) for chemicals tested as part of a mixture, and comparison to clearance rate for the single substance.

CHEMICAL	initial conc. (µM)	Slope	R^2	n	$CL_{int,in\ vivo}$ (SE)[a] (mL h^{-1} g $liver^{-1}$) tested in a mixture	$CL_{int,in\ vivo}$ (SE)[a] (mL h^{-1} g $liver^{-1}$) tested as single compound
$C_{10}SO_3$	0.2	-0.0111	0.97	15	113 (5) *	670 (36)
$C_{11}SO_3$	Not included					451 (19)
$C_{13}SO_3$	Not included					66 (5)
$C_{14}SO_3$	0.1	-0.0010	0.77	18	10 (1) [b]	n.s.
$C_{16}SO_3$	0.1	-0.0008	0.64	18	8 (2) [b]	n.s.
$C_{11}SO_4$	0.1	-0.0202	0.96	10	206 (14) *	415 (23)
$C_{13}SO_4$	0.1	-0.0042	0.97	18	43 (2)	59 (6)
C_{10}-1-LAS	0.1	-0.0050	0.96	18	51 (3) *	91 (5)
C_{12}-EO_4-SO_4	0.1	-0.0216	0.96	9	220 (18)	192 (30)
DOSS	0.3	-0.0021	0.76	18	21 (3)	21 (2)
BEHP	0.6	-0.0003	0.13	18	n.s.	n.s.

[a] $CL_{int,in\ vivo}$ values are reported as the calculated value with standard error between brackets (SE). The SE of $CL_{int,in\ vivo}$ was calculated from the SE of the fitted regression slope. The measured recovery scaling factor of 147.8 mg RT-S9 protein g $liver^{-1}$ was used.

[b] Using simulation analysis, the lowest detectable depletion rate constant for a 2h RT-S9 assay was determined to be approximately 0.14 h^{-1} (slope -0.001) for ionizable organic chemicals measured by LC-MS/MS (Chen et al. 2016). The slope values for $C_{14}SO_3$ and $C_{16}SO_3$ in the mixture were significantly different from 0 ($p<0.0001$), but close to this detection limit.

n.s. = depletion slope not significantly different from 0.

* Depletion rate tested in a mixture is significantly lower than that tested individually based on comparative analysis of the slopes with Graphpad PRISM 8. Details on statistics and starting concentrations for the single compound experiments are presented in Table S17.

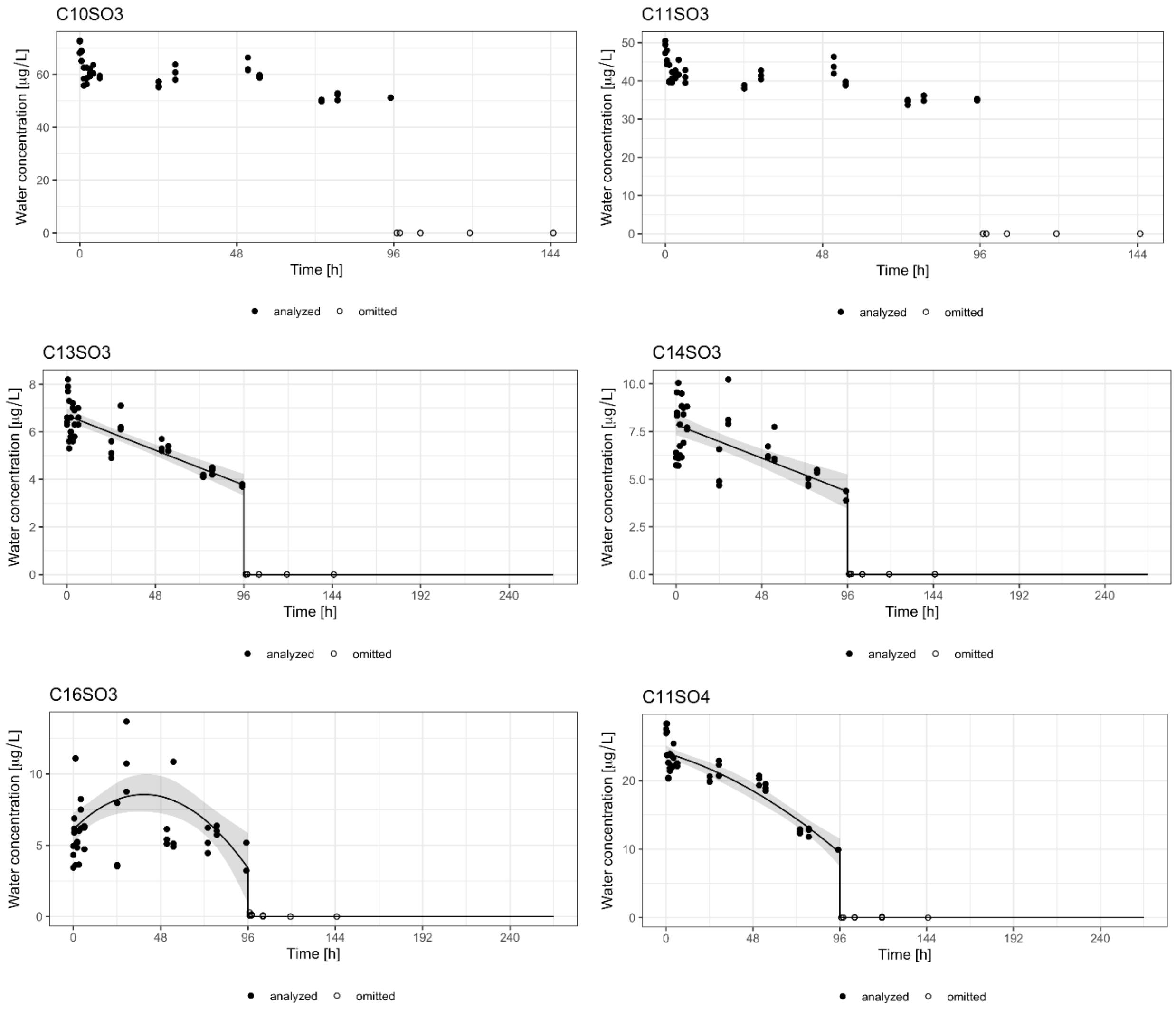

Figure S1: Surfactant concentrations in water during the exposure phase. The line shows modeled concentrations. Filled circles show data points included in the modeling.

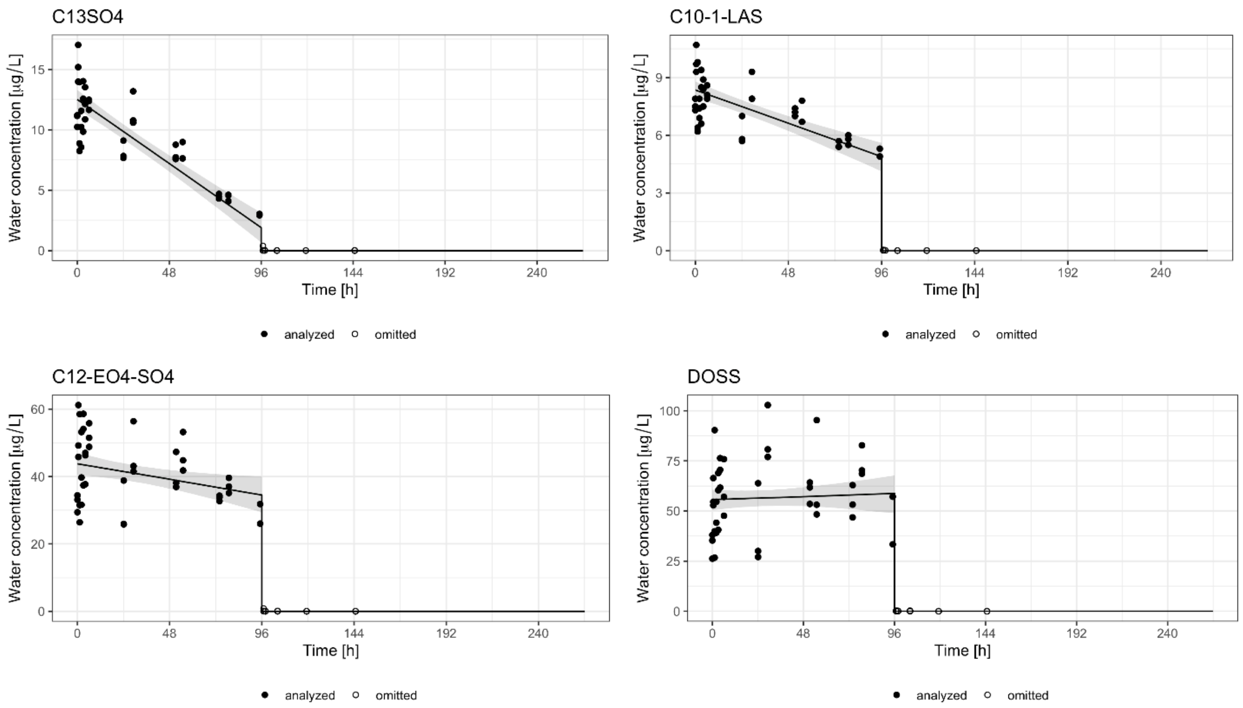

Figure S1 (continued): Surfactant concentrations in water during the exposure phase. The line shows modeled concentrations. Filled circles show data points included in the modeling.

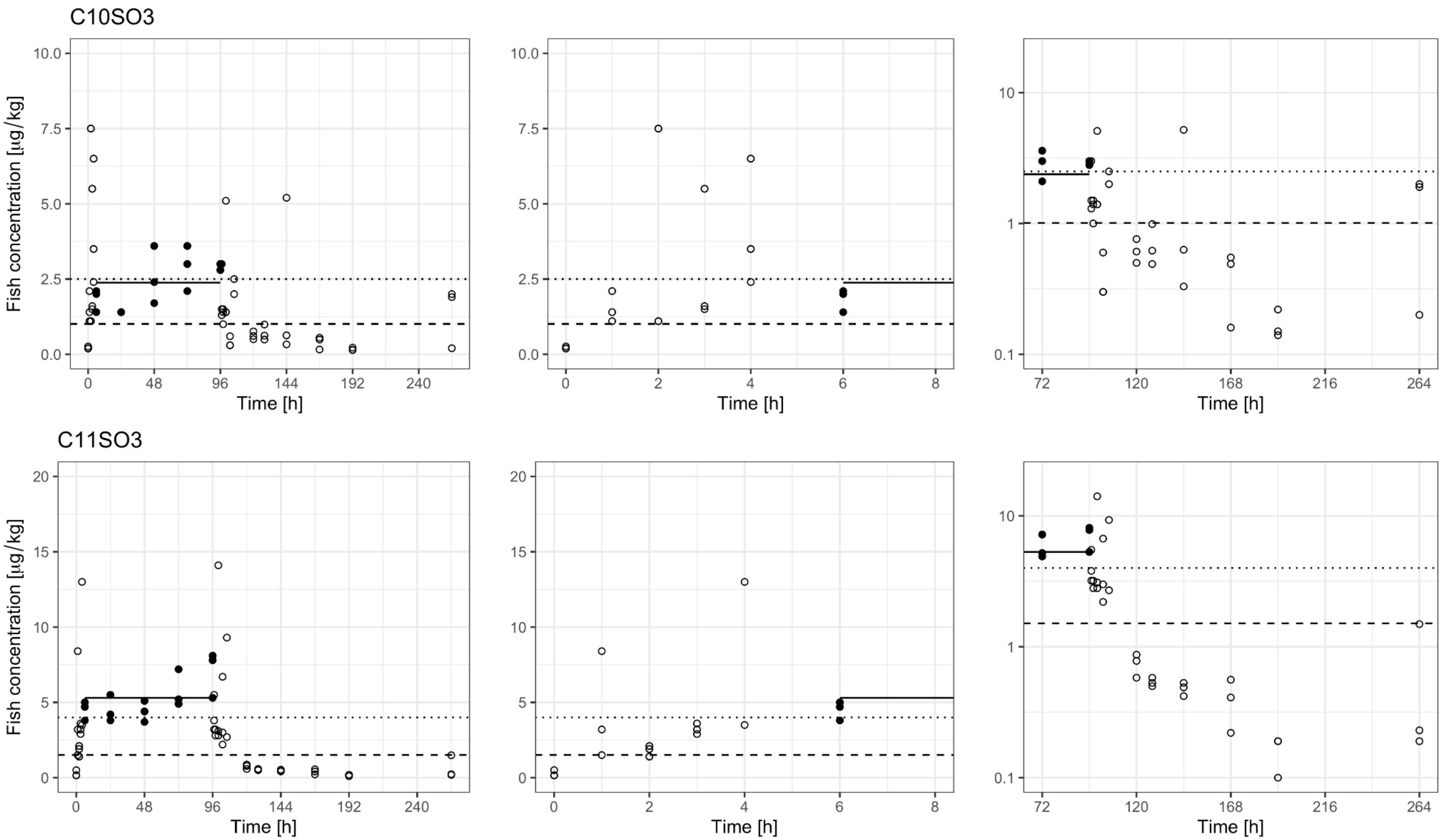

Figure S2: Plot of the surfactant concentrations in fish versus time. The dotted line shows the LOQ, while the dashed line shows the LOD. The solid line shows the fish concentration predicted from the steady state BCF. The filled circles show the measurements that were included in the estimation of the steady state BCF (all data between 6 h and 96 h). Left panel: whole experiment. Middle panel: first 6 h of the accumulation phase. Right panel: Elimination phase, semilogarithmic plot.

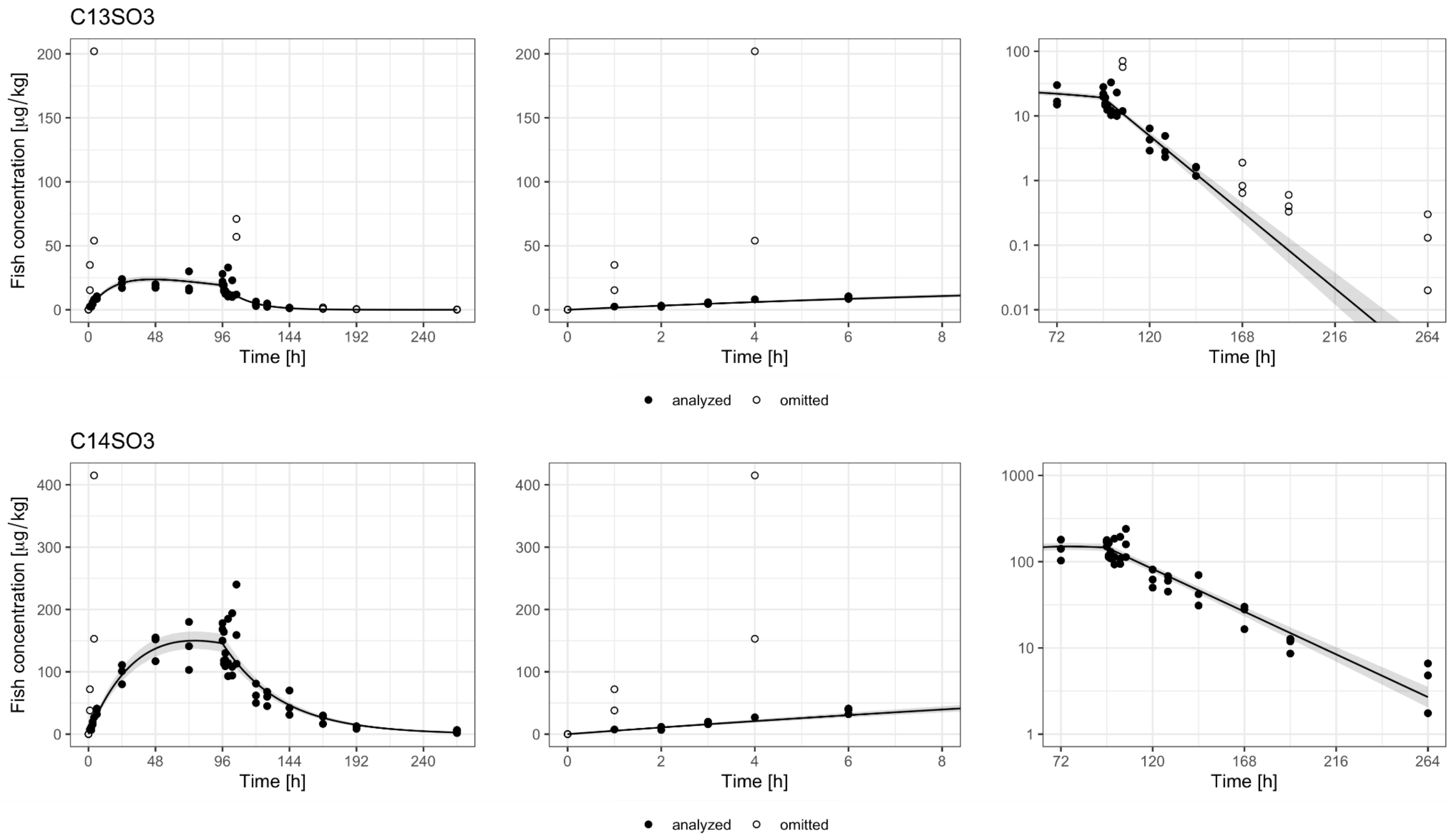

Figure S2 (continued): Plot of the surfactant concentrations in fish versus time. The solid line shows the fit of the one compartment model to the data. The filled circles show the measurements that were included in the model. The unfilled circles show measurements that were excluded: i) as outliers; ii) because at least one measurement at the time point was <LOQ. Left panel: whole experiment. Middle panel: first 6 h of the accumulation phase. Right panel: Elimination phase, semilogarithmic plot.

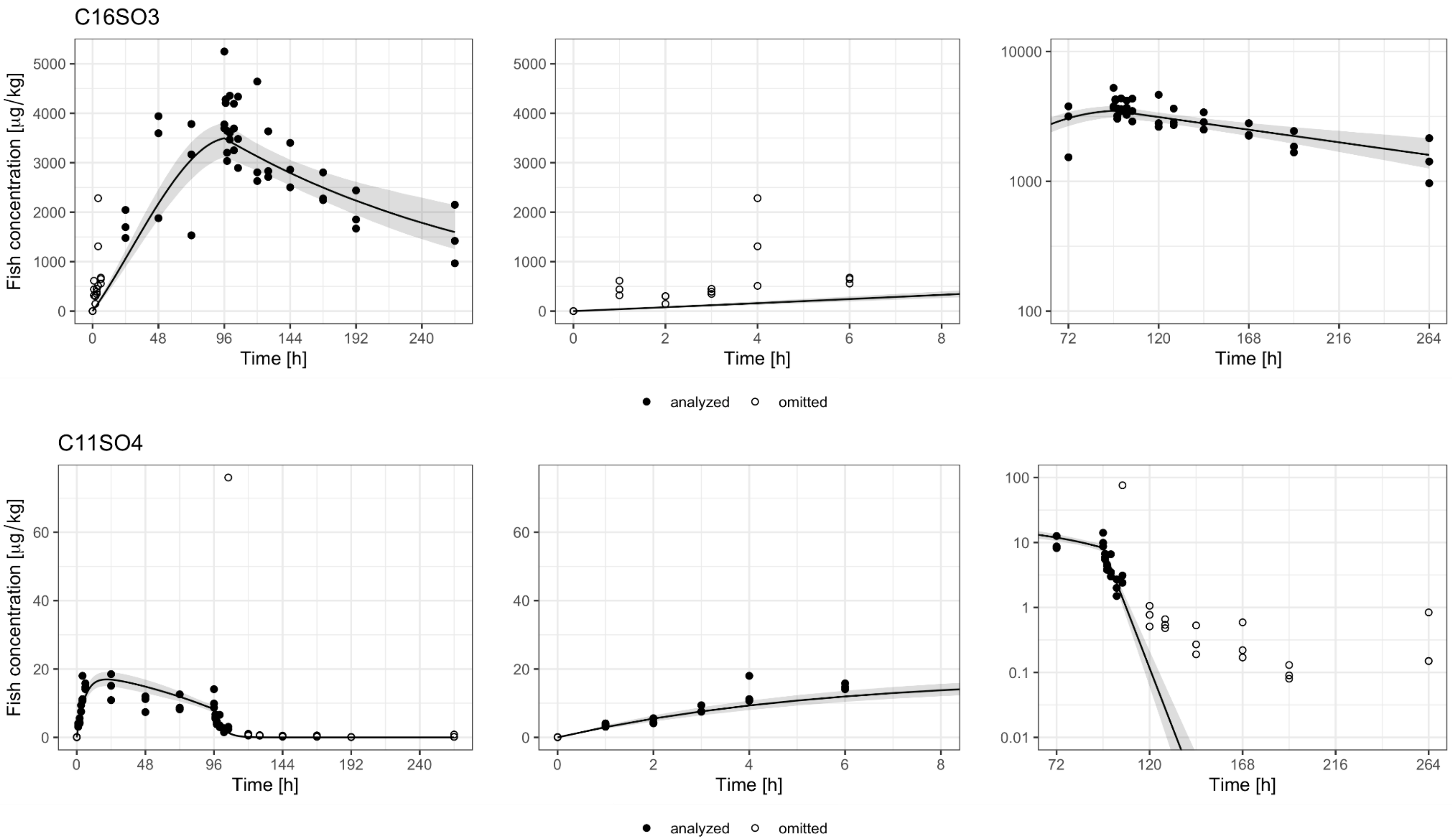

Figure S2 (continued): Plot of the surfactant concentrations in fish versus time. The solid line shows the fit of the one compartment model to the data. The filled circles show the measurements that were included in the model. The unfilled circles show measurements that were excluded: i) as outliers; ii) because at least one measurement at the time point was <LOQ; iii) first 6 h for $C_{16}SO_3$ (see manuscript). Left panel: whole experiment. Middle panel: first 6 h of the accumulation phase. Right panel: Elimination phase, semilogarithmic plot.

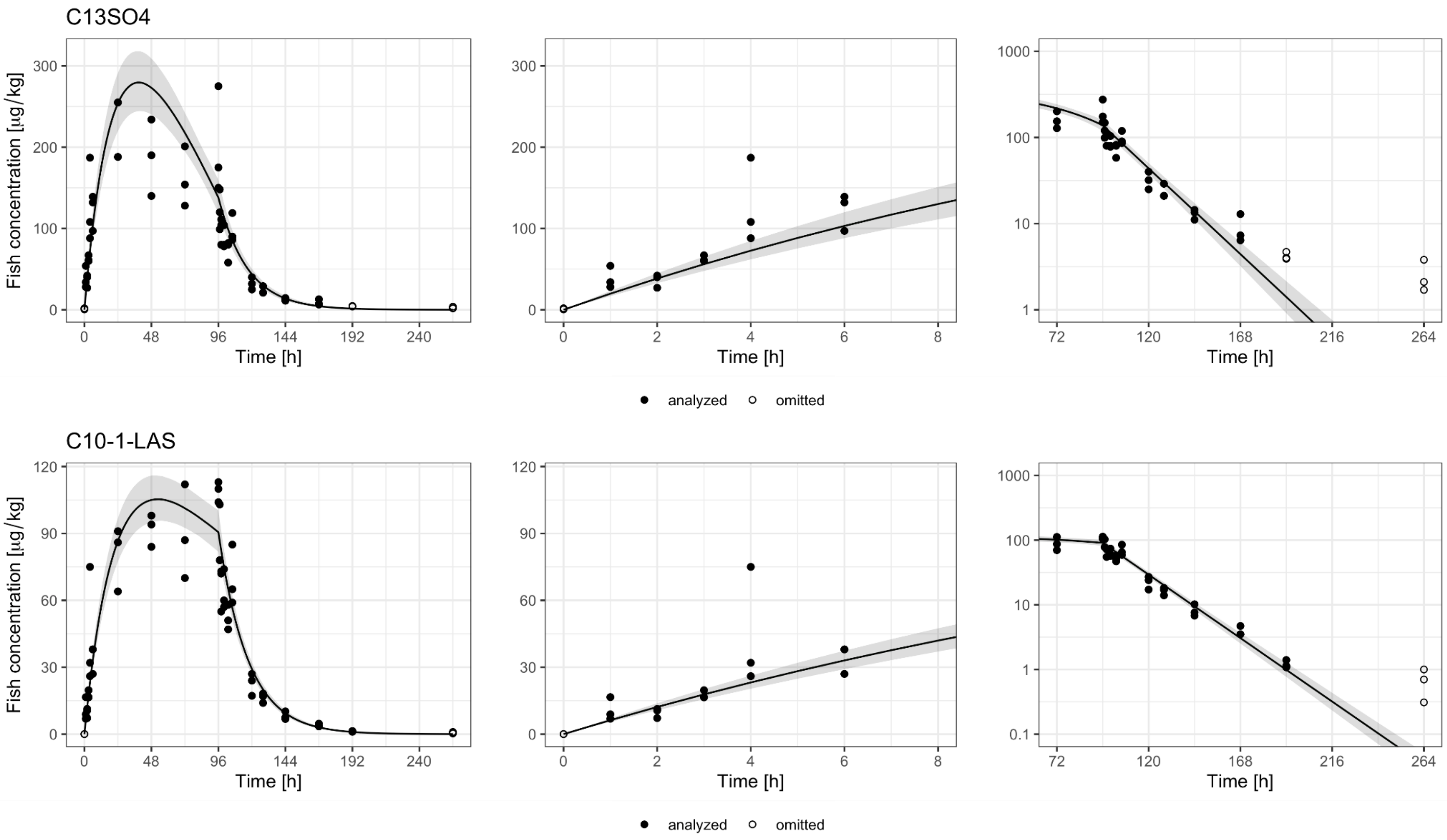

Figure S2 (continued): Plot of the surfactant concentrations in fish versus time. The solid line shows the fit of the one compartment model to the data. The filled circles show the measurements that were included in the model. The unfilled circles show measurements that were excluded: i) as outliers; ii) because at least one measurement at the time point was <LOQ. Left panel: whole experiment. Middle panel: first 6 h of the accumulation phase. Right panel: Elimination phase, semilogarithmic plot.

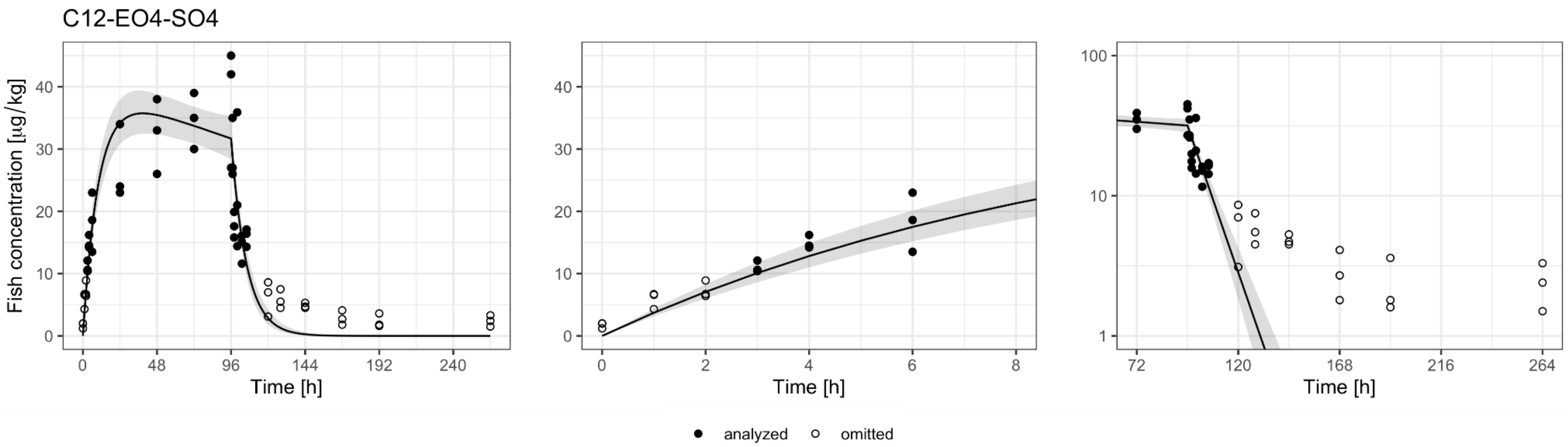

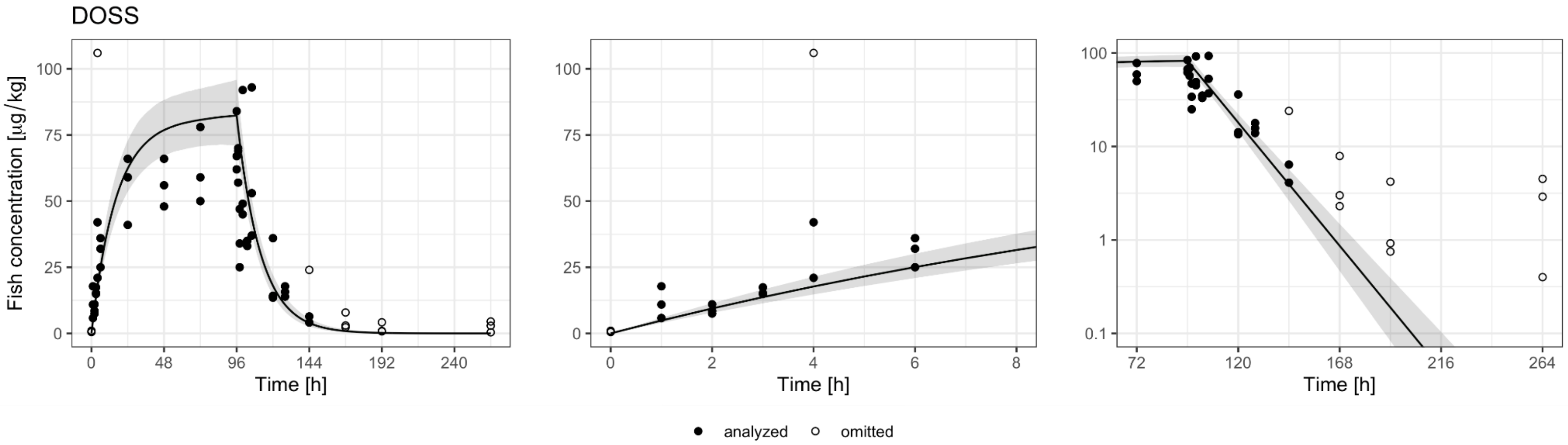

Figure S2 (continued): Plot of the surfactant concentrations in fish versus time. The solid line shows the fit of the one compartment model to the data. The filled circles show the measurements that were included in the model. The unfilled circles show measurements that were excluded: i) as outliers; ii) because at least one measurement at the time point was <LOQ. Left panel: whole experiment. Middle panel: first 6 h of the accumulation phase. Right panel: Elimination phase, semilogarithmic plot.

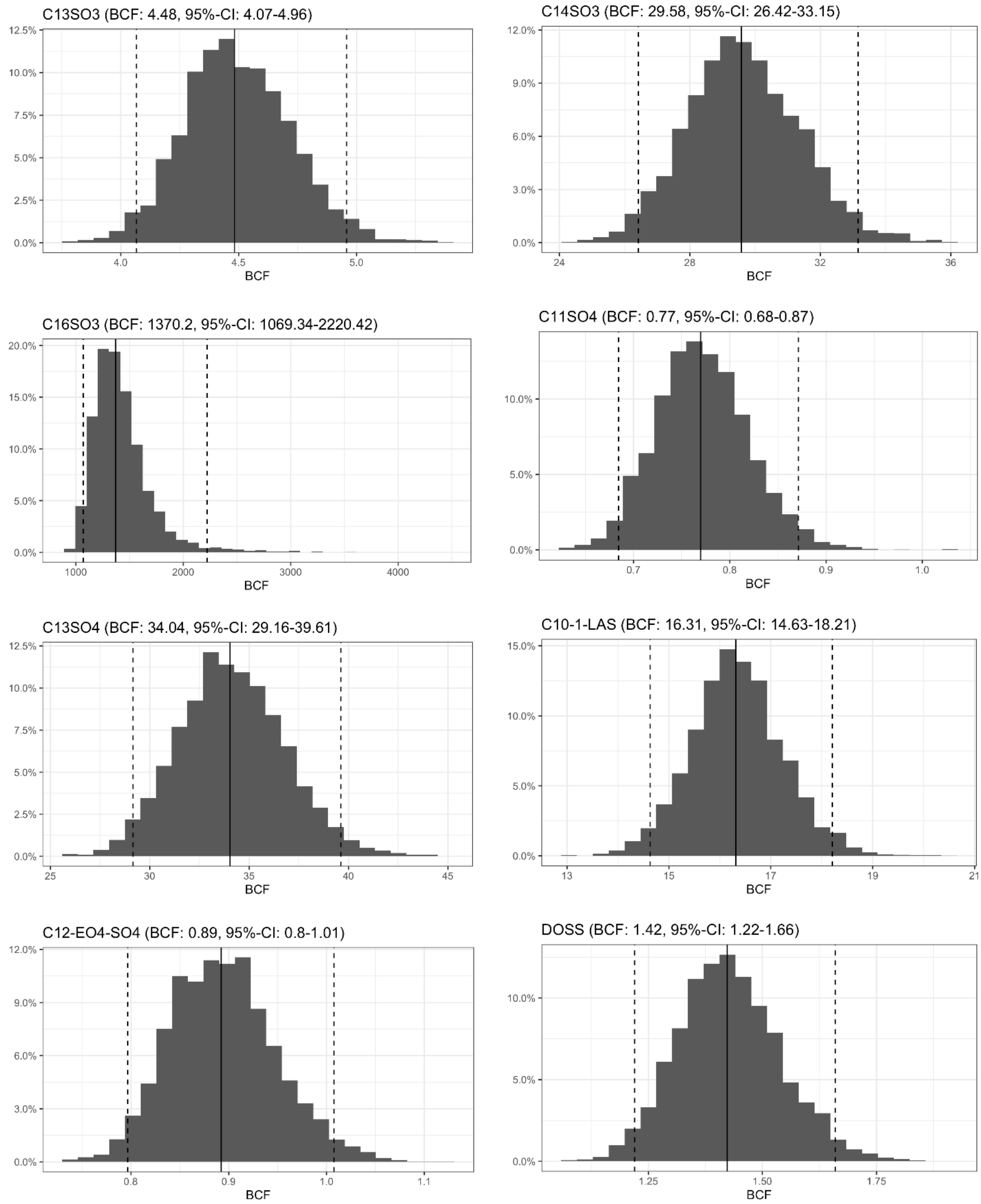

Figure S3: Statistical distribution of the estimate of BCF

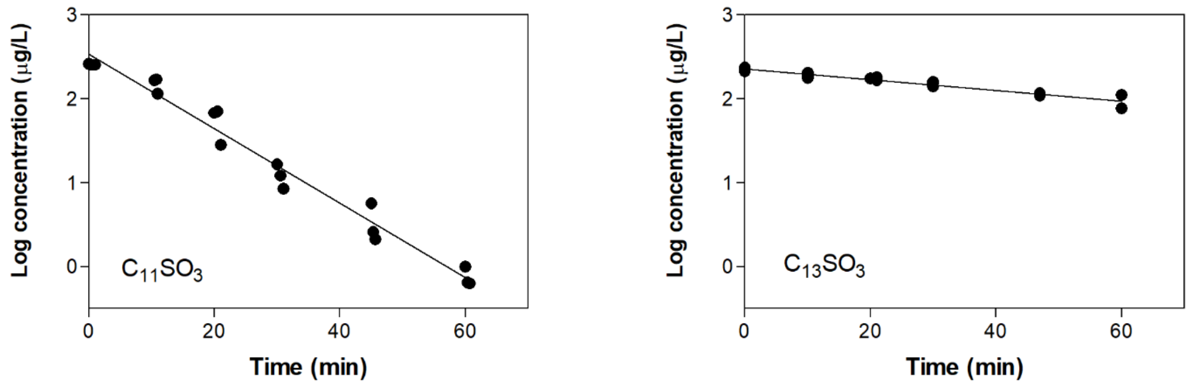

Figure S4: RT-S9 depletion data from triplicate series for $C_{11}SO_3$ (left plot) and $C_{13}SO_3$ (right plot) obtained in experiments with individual compounds. These two surfactants were not included in the mixture of anionic surfactants tested with active RT-S9 (Figure S4). No RT-S9 assay data were obtained with inactivated RT-S9, since Chen *et al*. (2014) found no losses with inactivated RT-S9 for homologue C_8SO_3 and $C_{12}SO_3$.

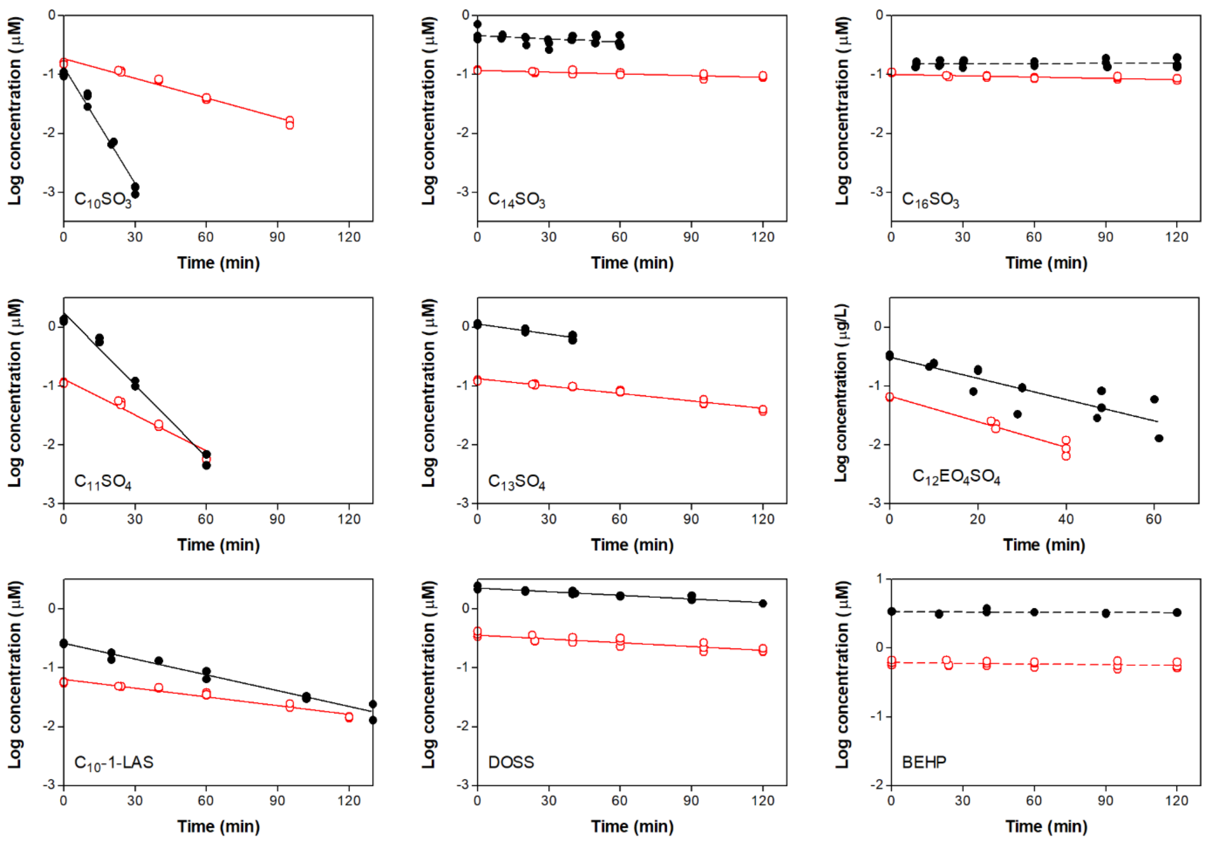

Figure S5: RT-S9 depletion data for anionic surfactants when tested in a mixture of 9 anionic surfactants (red open circles), in comparison to RT-S9 depletion data obtained for each individual compound alone with active RT-S9 (solid black dots). Solid lines have a slope significantly different from 0, broken lines have a slope not significantly different from 0 ($p > 0.05$).
